# Wemics: A Single‐Base Resolution Methylation Quantification Method for Enhanced Prediction of Epigenetic Regulation

**DOI:** 10.1002/advs.202308884

**Published:** 2024-03-28

**Authors:** Yi Liu, Jiani Yi, Pin Wu, Jun Zhang, Xufan Li, Jia Li, Liyuan Zhou, Yong Liu, Haiming Xu, Enguo Chen, Honghe Zhang, Mingyu Liang, Pengyuan Liu, Xiaoqing Pan, Yan Lu

**Affiliations:** ^1^ Key Laboratory of Precision Medicine in Diagnosis and Monitoring Research of Zhejiang Province Department of Respiratory Medicine, Department of Clinical Laboratory Sir Run Run Shaw Hospital and Institute of Translational Medicine Zhejiang University School of Medicine Hangzhou Zhejiang 310016 China; ^2^ Institute of Bioinformatics Zhejiang University Hangzhou 310058 China; ^3^ Department of Thoracic Surgery The Second Affiliated Hospital Zhejiang University School of Medicine Zhejiang University Hangzhou 310009 China; ^4^ Department of Physiology The University of Arizona Tucson AZ 85721 USA; ^5^ Department of Pathology Research Unit of Intelligence Classification of Tumor Pathology and Precision Therapy Chinese Academy of Medical Sciences Zhejiang University School of Medicine Hangzhou 310058 China; ^6^ Cancer center Zhejiang University Hangzhou 310058 China; ^7^ Department of Mathematics Shanghai Normal University Shanghai 200233 China; ^8^ Zhejiang Provincial Key Laboratory of Precision Diagnosis and Therapy for Major Gynecological Diseases Department of Gynecologic Oncology Women's Hospital and Institute of Translational Medicine Zhejiang University School of Medicine Hangzhou Zhejiang 310029 China

**Keywords:** DNA methylation, epigenetics, gene regulation, lung cancer, quantification methods, RRBS, RNA‐seq

## Abstract

DNA methylation, an epigenetic mechanism that alters gene expression without changing DNA sequence, is essential for organism development and key biological processes like genomic imprinting and X‐chromosome inactivation. Despite tremendous efforts in DNA methylation research, accurate quantification of cytosine methylation remains a challenge. Here, a single‐base methylation quantification approach is introduced by **we**ighting **m**ethylation of consecut**i**ve **C**pG **s**ites (Wemics) in genomic regions. Wemics quantification of DNA methylation better predicts its regulatory impact on gene transcription and identifies differentially methylated regions (DMRs) with more biological relevance. Most Wemics‐quantified DMRs in lung cancer are epigenetically conserved and recurrently occurred in other primary cancers from The Cancer Genome Atlas (TCGA), and their aberrant alterations can serve as promising pan‐cancer diagnostic markers. It is further revealed that these detected DMRs are enriched in transcription factor (TF) binding motifs, and methylation of these TF binding motifs and TF expression synergistically regulate target gene expression. Using Wemics on epigenomic‐transcriptomic data from the large lung cancer cohort, a dozen novel genes with oncogenic potential are discovered that are upregulated by hypomethylation but overlooked by other quantification methods. These findings increase the understanding of the epigenetic mechanism by which DNA methylation regulates gene expression.

## Graphical abstract

A single‐base methylation quantification approach by weighting methylation of consecutive CpG sites (Wemics) in genomic regions. By leveraging the information from neighboring CpG sites, which tend to have correlated methylation patterns, Wemics provides a reliable quantification of DNA methylation levels. Wemics quantification better predicts DNA methylation regulatory impact on gene transcription and identifies differentially methylated regions with more biological relevance.

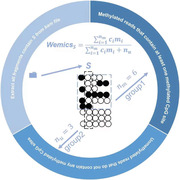

## 1 Introduction

Epigenetic mechanisms affect gene expression without changing its DNA sequence. As a critical component of epigenetics, DNA methylation plays a vital role in the development of organisms and is closely related to key processes such as genomic imprinting and X‐chromosome inactivation. It occurs predominantly on cytosine (C) and the methylated base is sometimes called the ‘fifth base of DNA’.^[^ ^1^ ^]^ One of the most common methylation processes is the covalent addition of a methyl group to a cytosine base.^[^ ^2^ ^]^ The occurrence of methylation on the genome is very common in mammals;^[^ ^3^ ^]^ most cytosines are methylated, primarily in the context of CpG sites.^[^ ^4^, ^5^ ^]^ CpG sites are regions of DNA where a cytosine nucleotide is followed by a guanine nucleotide in the linear sequence of bases and the two nucleotides are joined with the phosphodiester bond. Gene promoters are rich in CpG sites, and the methylation status of CpG in gene promoter often affects its gene expression. Aberrant DNA methylation modification is frequently involved in various diseases including cancer.^[^ ^3^, ^6^ ^]^ For instance, cancer tissues often exhibit two opposite changes in their DNA methylation patterns.^[^ ^7^ ^]^ The increased level of local DNA hypermethylation in CpG islands (CGI) usually leads to the repression of tumor suppressor genes.^[^ ^8^, ^9^ ^]^ On the contrary, the global hypomethylation on the genome induces genome instability and triggers structural variations.^[^ ^10^, ^11^ ^]^

Genome‐wide DNA methylome can be obtained by various methods, such as those based on restriction enzymes, affinity enrichment, or bisulfite conversion.^[^ ^12^ ^]^ Reduced representation bisulfite sequencing (RRBS) is currently one of the most commonly used and efficient method for profiling genome‐wide DNA methylation.^[^ ^13^, ^14^ ^]^ It uses restriction enzymes to enrich DNA segments with a high CpG content and regulatory potentials, and then uses bisulfite treatment to convert cytosine residues to uracil without affecting 5‐methylcytosine residues, followed by next‐generation sequencing of these bisulfite‐converted DNA fragments.

Proper quantification of RRBS data is the most important first step in studying DNA methylation. The methylation level of a genomic region has long been quantified by $\frac{C}{C+T}$ (i.e., the average proportion of methylated cytosines in a genomic region, termed as Meanm). Recently, CHALM was proposed to quantify the promoter methylation as the percentage of reads with at least one methylated CpG site to the total reads mapped to a given promoter region.^[^ ^15^ ^]^ However, CHALM does not distinguish between reads containing different methylated CpG sites and information on the location of these sites. Another important quantification method is Concurrence of Active Methylation and De‐methylAtion (CAMDA), which instead focuses on the unmethylated CpGs in partially methylated reads.^[^ ^16^ ^]^ Although the regulatory role of promoter methylation in gene transcription has been well known, the correlation between methylation and gene expression levels has been weak.^[^ ^17^, ^18^, ^19^ ^]^ A good DNA methylation quantification method should reflect a strong negative correlation between gene promoter methylation level and its gene expression, thereby increasing our understanding of epigenetic mechanism by which DNA methylation regulates gene expression.

**Figure 1:**
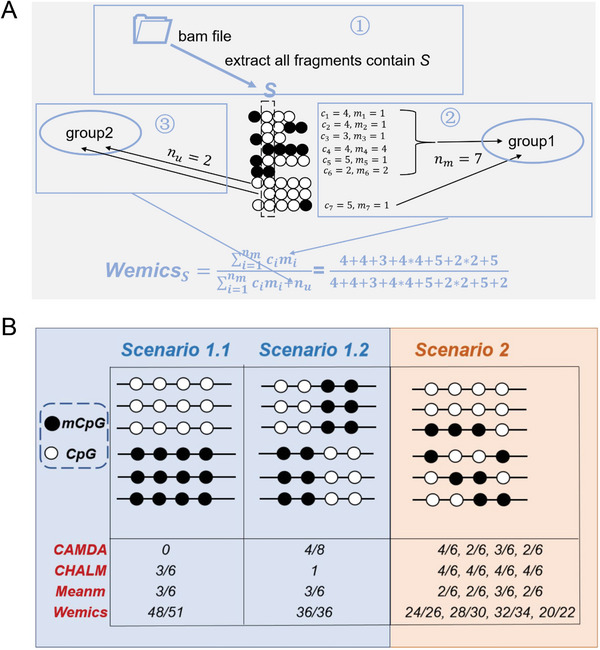
Main workflow of Wemics for quantifying DNA methylation in RRBS data. A) Schematic diagram of Wemics quantification of DNA methylation at CpG sites. B) Numeric illustration of DNA methylation levels at CpG sites quantified by different methods. Black circles represent methylated CpG sites and blank circles represent unmethylated CpG sites. A cluster of circles in a row represents a sequenced read which was retrieved from the BAM file of a RRBS library. Group 1 consists of a set of reads that contain at least one methylated CpG site, i.e., methylated reads. Group 2 consists of a set of reads that do not contain any methylated CpG sites, i.e., unmethylated reads. All reads in Groups 1 and 2 contain CpG site *S*. *c_i_* represents the number of CpG sites in the *i*‐th read, *m_i_* represents the weight reflecting the co‐methylation adjacent to the CpG site S in the *i*‐th read.

In this study, we introduced a single‐base resolution methylation quantification method by **we**ighting methylation of consecutive CpG sites (Wemics) in genomic regions (**Figure** **1**). Wemics reveals cell heterogeneity in DNA methylation by distinguishing methylated reads, unmethylated reads and partially methylated reads in bulk sequenced cells. It provides reliable quantification of DNA methylation levels by borrowing information from consecutive CpG sites as there is a strong dependency of DNA methylation among consecutive CpG sites. To illustrate the utility of Wemics, we generated 144 RRBS libraries from 77 tumor tissues and their matched adjacent normal tissues of non‐small cell lung cancer (NSCLC) patients, of which 55 pairs of NSCLC samples have corresponding RNA‐seq data. Wemics quantification of DNA methylation better predicts its impact on gene transcription and identifies differentially methylated regions (DMRs) with more biological relevance.

## 2 Results

### 2.1 Overview of Wemics

Wemics is a single‐base resolution method for quantifying DNA methylation in RRBS data. Wemics not only considers methylation status on its own CpG site and whether the sequenced read in which it is located contains methylated CpG sites, but also weights co‐methylation of its adjacent consecutive CpG sites (Figure 1A). It provides reliable quantification of DNA methylation levels by borrowing information from consecutive CpG sites as there is a strong dependency of DNA methylation among consecutive CpG sites.

Wemics reveals cell heterogeneity in DNA methylation patterns by distinguishing methylated reads, unmethylated reads, and partially methylated reads in bulk sequenced cells. For example, in both scenarios 1.1 and 1.2 (Figure 1B), although 50% of the CpG sites in these two regions are methylated, the distribution of methylated sites on their sequenced reads is quite different. When the Meanm method was used to quantify the methylation intensity of CpG sites, it was not possible to distinguish the different distribution of methylated CpG sites in these two regions since the methylation of each of CpG sites was quantified as 0.5 in both scenarios. While the other three methylation quantification methods can well distinguish the difference in methylation intensity between these two scenarios, with CAMDA showing the largest difference, Wemics the smallest, and CHALM in between. However, in scenario 2, Wemics is the only quantification method that could distinguish between different methylation intensities of four CpG sites in the sequenced read. These results showed that the quantification of Wemics makes the differences in methylation levels between different CpG sites more prominent and facilitates subsequent detection of differentially methylated regions in the samples.

### 2.2 Lung Cancer RRBS and RNA‐Seq Datasets

To comprehensively assess the performance of different methods for quantifying DNA methylation, we generated 144 RRBS libraries from 77 tumor tissues and their matched adjacent normal tissues from NSCLC patients (Table S1, Supporting Information). On average, each RRBS library yielded ≈28 million reads with 5 cytosines per read (Figure S1A, Supporting Information), and the average bisulfite conversion rate was 99.7% (Table S2, Supporting Information). 2,824,663 CpG sites were detected with at least 5X in more than 80% of samples. These NSCLC tumor tissues and their matched adjacent normal tissues were clearly separated, showing two distinct methylation patterns with a large variability in tumor tissues (Figure S1B,C, Supporting Information). It was observed that the majority of sequence reads were either fully methylated or unmethylated, with only a small fraction of them partially methylated (Figure S1D, Supporting Information). Since each read likely represents a single cell within the sequenced bulk cells, gene promoters from which most reads were sequenced are highly heterogeneous.

In these RRBS libraries, 55 tumor tissues and their matched adjacent normal tissues also had corresponding RNA‐seq data (Table S2, Supporting Information). Each RNA‐seq library generated approximately 43 million reads with average mapping rate of 94.2% (Table S3, Supporting Information). As expected, there are two distinct gene expression patterns between lung tumor tissues and adjacent normal lung tissues (Figure S1E,F, Supporting Information).

### 2.3 Wemics Quantification of DNA Methylation Better Predicts its Impact on Gene Transcription

**Figure 2:**
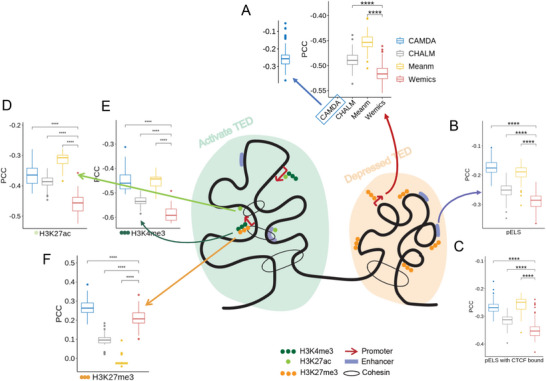
Wemics quantification of DNA methylation is a better predictor of its impact on gene transcription. A) Correlation between gene expression and its promoter methylation quantified by different methods. B,C) Correlation between gene expression and its enhancer methylation quantified by different methods. Two types of enhancers were analyzed: enhancer‐like signatures (pELS) without CTCF binding (B), and pELS bound by CTCF (pELS_CTCFbound) (C). D–F) Correlation between histone mark signal intensity and promoter methylation quantified by different methods in promoter CGIs. Three types of histone marks were analyzed: H3K27ac (D), H3K4me3 (E), and H3K27me3 (F). Pearson correlation coefficients (PCC) with p‐values were calculated; ^****^ *p* < 0.001.

Methylation of gene promoter can regulate gene transcription by interfering with transcription factor (TF) binding, recruiting Methyl‐CpG‐binding domain (MBD) proteins, and altering chromatin structure, often resulting in repression of the expression of genes associated with the promoter.^[^ ^7^, ^20^ ^]^ Therefore, a good DNA methylation quantification method should reflect a strong negative correlation between gene promoter methylation level and its gene expression. Wemics weights co‐methylation of consecutive CpG sites to quantify DNA methylation at single‐base resolution in genomic regions (Figure 1). We compared our new method with the traditional mean methylation (refer to Meanm) and the two recently developed methods, CHALM and CAMDA, in our large NSCLC dataset (**Figure** **2**).

We first applied these four different methods (Wemics, Meanm, CHALM, and CAMDA) to quantify methylation levels on gene promoters in our NSCLC RRBS data, and then performed Pearson correlation analysis of DNA methylation level with its gene expression in the corresponding NSCLC RNA‐seq data. As expected, methylation levels of gene promoters were significantly negatively correlated with their gene expression, with mean Pearson correlation coefficients (PCC) of −0.52, −0.45, −0.49, and −0.28 for Wemics, Meanm, CHALM, and CAMDA, respectively (Figure 2A). There were no differences in Pearson correlations between lung tumors and adjacent normal tissues except for CAMDA (Figure S2A, Supporting Information). Furthermore, all genes showed a nadir of methylation levels on their TSS; the nadir was more pronounced for genes with higher expression (Figure S2B, Supporting Information). The upstream and downstream of the TSS showed progressively increasing methylation levels as their distance from the TSS increased (Figure S2B,C, Supporting Information).

Enhancers are important cis‐regulatory elements that work in concert with promoters to regulate gene transcription. Thus, two types of cis‐regulatory elements proximal to TSS were analyzed, enhancer‐like signatures (pELS) without CTCF binding and pELS bound by CTCF (pELS_CTCFbound). Likewise, the methylation levels of these cis‐regulatory elements were negatively correlated with the expression levels of the genes they regulated; pELS_CTCFbound had a stronger negative correlation than pELS. Of the four quantification methods, Wemics yielded the strongest negative PCC in either pELS or pELS_CTCFbound (Figure 2B,C).

In addition, histone modification and DNA methylation are highly interactive in gene regulation, with synergistic or antagonistic effects on gene expression.^[^ ^21^, ^22^ ^]^ H3K27ac and H3K4me3 are often associated with gene activation, and their signal intensities are negatively correlated with DNA methylation, whereas H3K27me3 is a repressive epigentic mark of gene expression and positively correlates with DNA methylation.^[^ ^23^, ^24^ ^]^ We thus assessed the relationship between intensities of histone marks in promoter CGIs and DNA methylation levels quantified by different methods. The methylation levels around H3K4me3 and H3K27ac peaks showed a largely symmetrical distribution pattern from low to high (Figure S2D,E, Supporting Information), while the methylation level was highest near the H3K27me3 peak and gradually decreased along its sides (Figure S2F, Supporting Information). Methylation quantified by Wemics was more negatively associated with H3K27ac (*r* = −0.45) and H3K4me3 (*r* = −0.58) than other methods (Figure 2D,E). The correlation coefficient of Wemics‐quantified methylation and H3K27me3 (*r* = 0.21) was slightly lower than that of CAMDA‐quantified methylation and H3K27me3 (*r* = 0.27) (Figure 2F).

Although H3K27me3 marks are commonly associated with gene repression, unlike H3K9me3, which remains consistently silenced, H3K27me3 permits gene activation through transcription factor binding under specific circumstances.^[^ ^25^, ^26^ ^]^ This complexity is evident as H3K27me3 acts as a gene repression signal for most genes, yet a subset can still be activated through alternative pathways, leading to observed high gene expression despite the H3K27me3 modification. Given that promoter CGIs represent approximately one‐third of all genes in our data, we extended the analysis to all genes on the genome. The resulting correlation coefficient between Wemics‐quantified methylation and H3K27me3 reached its highest value at 0.22 (Figure S2G, Supporting Information). Similar trends were observed between Wemics‐quantified methylation and H3K27ac or H3K4me3 when considering all genes on the genome. Taken together, Wemics quantification of DNA methylation better predicts its effect on gene transcription than other quantitative methods.

### 2.4 Comparison of Unpaired and Paired Metilene in Identifying DMRs Between Tumors and their Matched Normal Tissues

Metilene, a commonly used de‐novo DMR detection tool, applies a circular binary segmentation algorithm to search for genomic regions that maximize methylation differences between two unpaired groups using the Mann‐Whitney U test.^[^ ^27^ ^]^ To make it applicable for the analysis of tumor‐ and normal‐matched RRBS data, we therefore replaced the Mann‐Whitney U test by the Wilcoxon signed‐rank test in metilene. We employed the metilene (unpaired) and its modified version (paired) to identify DMRs in our 77 pairs of NSCLC tumors and adjacent normal tissues.

**Figure 3:**
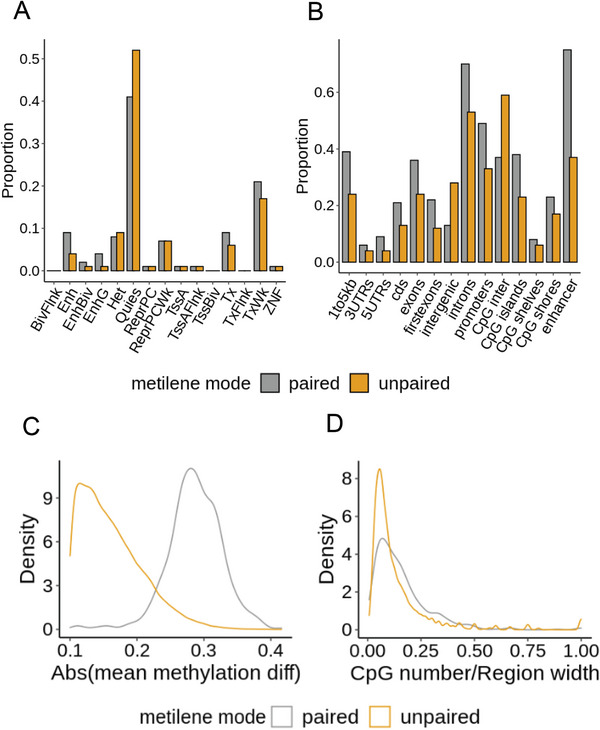
Comparison of DMRs detected by the unpaired and paired metilene. A) Proportion of DMRs with different chromatin states. B) Proportion of DMRs with different genomic features. C) Distribution of absolute methylation difference in DMRs. D) Distribution of CpG densities in DMRs. A modified Metilene with Wilcoxon signed‐rank test (paired metilene) and original metilene (unpaired metilene) were used to detect DMRs under Meanm quantification method. Grey bars or lines represent the results of the paired metilene, and orange bars or lines represent the results of the unpaired metilene.

We compared DMRs identified by the two methods in terms of chromatin states and genomic locations (Table S4, Supporting Information). DMRs identified by the paired metilene accounted for a higher proportion of regions with chromatin states such as Tx, TxWK, Enhg, and Enh, which are key regions for gene regulation, while DMRs identified by the unpaired metilene had a higher proportion of quiescent regions (i.e., Quies) that show very little signal for any of functional genomic features (**Figure** **3**A). Furthermore, DMRs identified by the paired metilene were more frequently presented in enhancers, exons, first exons, and promoters than those identified by the unpaired metilene (Figure 3B). The overlapped and unique DMRs detected by paired and unpaired metilene are shown in Figure S3 (Supporting Information).

In the regions of DMRs detected by the paired metilene, the average methylation difference between tumors and their matched normal tissues was ≈0.3. In contrast, the unpaired metilene detected an average methylation difference of 0.1 across DMR regions (Figure 3C). A recent study showed that metilene had poor precision when the mean methylation difference between the two groups was low.^[^ ^28^ ^]^ Furthermore, the regions of DMRs identified by the paired metilene had a higher CpG density than those identified by the unpaired metilene, reminiscent of higher regulatory potential (Figure 3D). These data suggested that the paired metilene appears to identify more biologically relevant de‐novo DMRs than the unpaired metilene in tumor and normal matched samples.

### 2.5 Wemics Captures more DMRs Distributed in Functional Regulatory Regions

**Figure 4:**
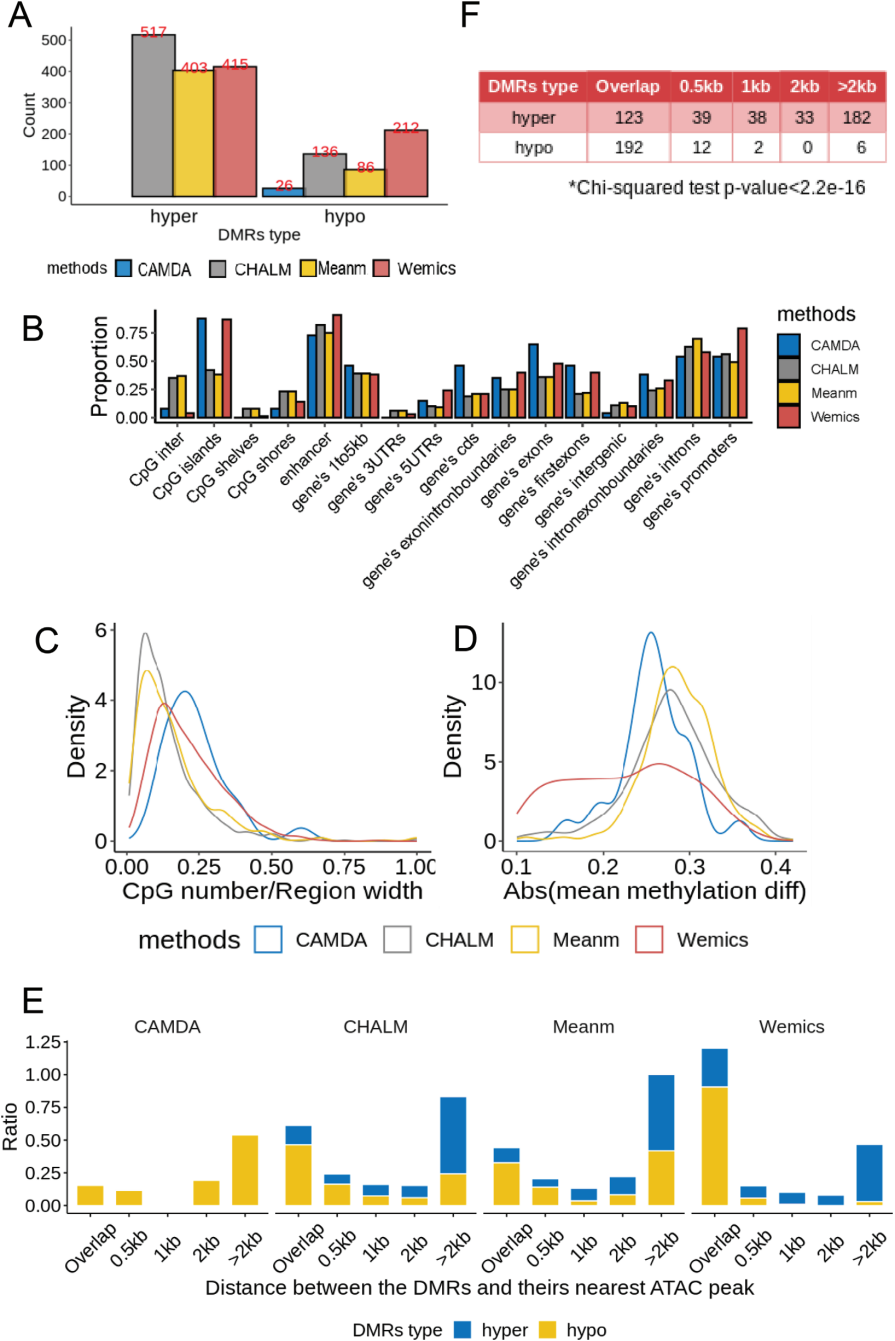
DMRs detected under Wemics methylation quantification were distributed more frequently in functionally regulatory regions. A) Number of DMRs identified under different methylation quantification methods. B) Comparison of genomic features of DMRs identified under different methylation quantification methods. C) Distribution of CpG densities in DMRs identified under different methylation quantification methods. D) Distribution of absolute methylation differences in DMRs identified under different methylation quantification methods. E) Distances between DMRs identified by different methylation quantification methods and their nearby ATAC peaks. F) Number of Wemics‐quantified DMRs at different distances from nearby ATAC peaks (top right panel).

Next, we applied the paired metilene to identify DMRs between tumors and their matched normal tissues of our NSCLC cohort using methylation levels quantified by the four different methods (Table S5, Supporting Information). As a result, the number of DMRs detected by the four quantification methods varied greatly (**Figure** **4**A). CAMDA detected the least number of DMRs with only 26 Hypo‐DMRs (hypomethylated regions in tumor tissues as compared with adjacent normal tissues) but no Hyper‐DMRs (hypermethylated regions in tumor tissues as compared with adjacent normal tissues). CHALM detected the largest number of Hyper‐DMRs (517) and a modest number of Hypo‐DMRs (136). Wemics detected a relatively balanced number of DMRs, including 415 Hyper‐DMRs and 212 Hypo‐DMRs, respectively. Meanm detected a total of 489 DMRs, including 403 Hyper‐DMRs and 86 Hypo‐DMRs.

To elucidate the overlap and distinction among DMRs detected by the four different methods, we performed a two‐by‐two comparison of DMRs detected under each method. The results show that the overlap between any two approaches was less than 50%, which suggests that each quantification method has its own uniqueness (Figure S4A, Supporting Information). Wemics DMRs exhibited the highest mean depth compared to the other methods (Figure S4C, Supporting Information). Additionally, in considering the simultaneous detection of DMRs by all four methods, we noted a notable similarity between CHALM and Meanm, identifying 222 common regions (Figure S4B, Supporting Information). Moreover, our examination of unique Wemics DMRs revealed consistently higher mean depth when compared to other methods (Figure S4D, Supporting Information).

Hypo‐DMRs in gene promoters are often closely associated with increased expression of oncogenes, which is one of the key mechanisms of cancinogenesis.^[^ ^29^ ^]^ Wemics has a stronger ability of finding Hypo‐DMRs than the other three methods. In contrast, relatively small numbers of Hypo‐DMRs were detected under other methylation quantification methods, and using these methods to quantify DNA methylation may overlook key oncogenes in NSCLC.

Compared with other quantification methods, Wemics detected a higher proportion of DMRs distributed on functionally important regulatory regions such as CpG islands and promoters (Figure 4B). Among DMRs identified by Wemics, they had higher CpG density (Figure 4C) and tended to have smaller methylation differences between tumors and adjacent normal tissues (Figure 4D). In addition, we explored the relationship between chromatin accessibility and methylation using ATAC‐seq data of A549 cell line. In NSCLC, 51.3% of chromatin peaks were located in intergenic regions, 33.19% were located in gene bodies, and only 15.51% of peaks were located in gene promoters (Figure S5, Supporting Information). Hypo‐DMRs were generally closer to open chromatin regions, while hyper‐DMRs were mostly associated with closed chromatin (Figure 4E). This suggests that methylation levels within the exposed DNA region are typically lower, thereby facilitating the binding of various functional proteins to this region and thus activating transcription of target genes. The relationship between methylation and chromatin openness in Wemics‐detected DMRs supports the above idea better than other methylation quantification methods. Specifically, more than 90% of Hypo‐DMRs detected by Wemics overlaped with the chromatin open region identified by ATAC‐seq, whereas only 29.60% hyper‐DMRs overlapped with ATAC‐seq peaks (Figure 4E). Furthermore, under Wemics quantification, Hyper‐DMRs and Hypo‐DMRs differed significantly in their distribution patterns with respect to the distance to chromatin open regions (p‐value <2.2e‐16, Figure 4F).

### 2.6 Wemics‐Detected DMRs have more Potential to Regulate their Gene Expression

**Figure 5:**
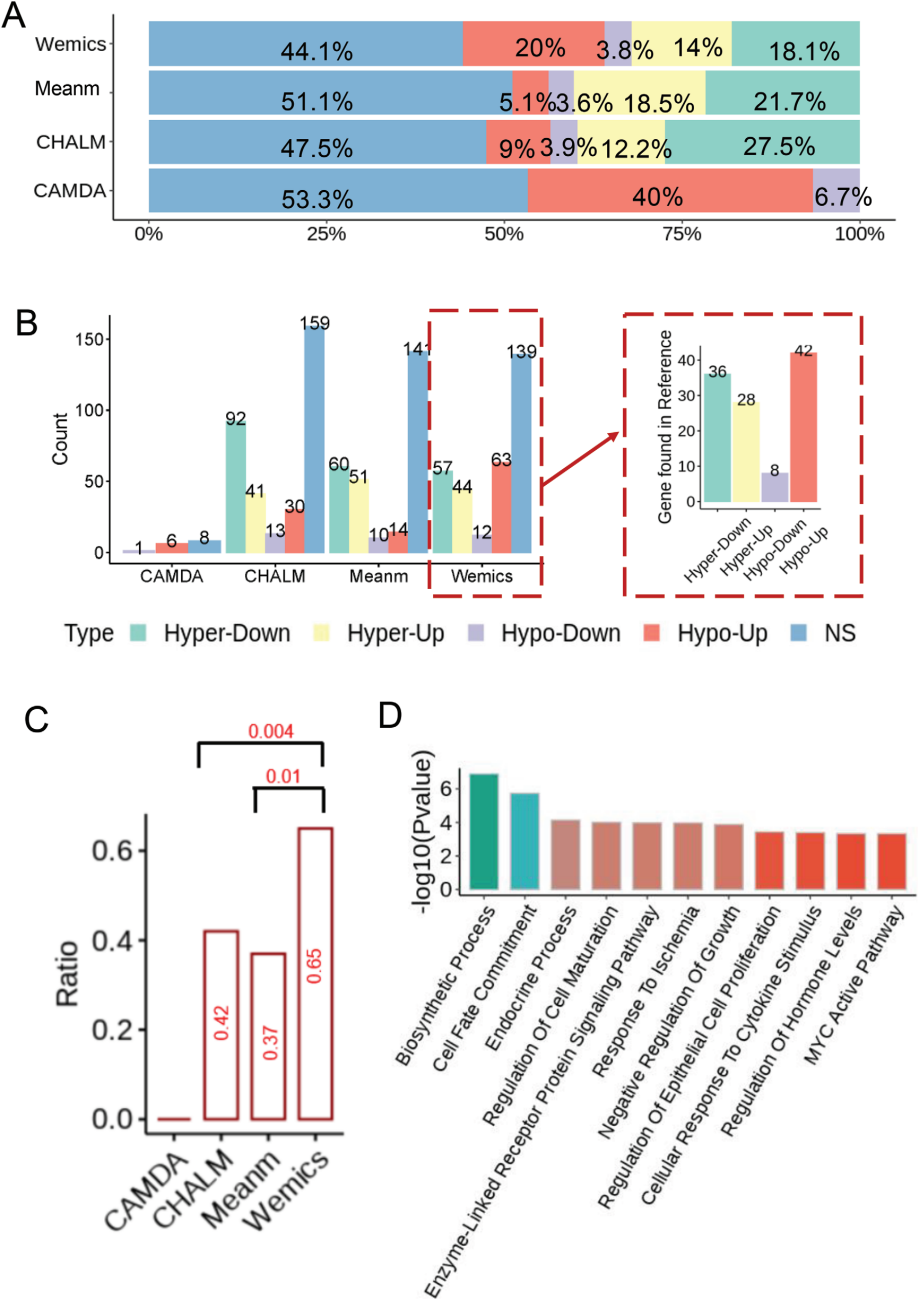
DMRs detected under Wemics methylation quantification have more potential to regulate their gene expression. A) Proportion of different types of DEGs associated with DMR detected under different methylation quantification methods. B) Number of different types of DEGs associated with DMR detected under different methylation quantification methods. Number of genes associated with Wemics‐quantified DMRs, which have been implicated in lung cancer in previous studies, is shown in the right panel. C) Proportion of DMRs associated with Hyper‐Down genes located in promoters. P‐values were calculated by two proportion z‐test. D) Pathways enriched for Hyper‐Down and Hypo‐UP genes under Wemics methylation quantification. Geometric test was used to assess whether there was a significant enrichment of genes for a particular pathway.

Then, we determined whether genes containing these DMRs (including Hyper‐ and Hypo‐DMRs) were differentially expressed between lung tumors and adjacent normal tissues, i.e., DEGs, which were further classified as Up‐ and Down‐regulated in tumors compared with adjacent normal tissues (**Figure** **5**A). Therefore, according to the changes in their expression and methylation levels, these DMRs‐associated genes could be classified into five categories: Hyper‐Down, Hyper‐Up, Hypo‐Down, Hypo‐Up, and NS (not significantly differentially expressed between tumors and adjacent normal tissues). Among them, Hyper‐Down and Hypo‐Up lists represent the well‐known inverse relationship between gene expression and its associated DNA methylation level, thereby being the leading candidates for methylation‐induced driver genes. We found that Wemics had detected more unique Hyper‐Down and Hypo‐Up genes compared to the other three quantification methods (Figure S6A,B, Supporting Information). Interestingly, Hyper‐Down genes exhibit more commonality across different methods, while Hypo‐Up genes are predominantly unique. Under a specific quantification method, the unique DMRs, domonating Hyper‐Down and Hypo‐Up genes, showcased the highest absolute level of methylation difference between tumor and normal groups, rendering them more likely to be detected as DMRs (Figure S7C, Supporting Information).

Wemics‐detected DMRs contained the largest proportion of DEGs (55.9%), with Hypo‐Up (20.0%) and Hyper‐down (18.1%) genes accounting for the large majority of DEGs, whereas for CHALM and Meanm, Hyper‐Down and Hyper‐Up genes were the two largest DEG lists. More than 65% of the DEGs containing Wemics‐detected DMRs have been implicated in lung cancer in previous studies through MalaCards (https://www.malacards.org/)^[^ ^30^ ^]^ (Figure 5B and Table S6, Supporting Information). For example, Hypo‐Up genes such as ODC1,^[^ ^31^ ^]^ BCL11A,^[^ ^32^ ^]^ CDCA7,^[^ ^33^ ^]^ and TRIM2,^[^ ^34^ ^]^ detected under Wemics quantification, were shown to play important roles in the development and progression of NSCLC. This finding represents the highest reported ratio of Hyper‐Down and Hypo‐Up genes across the four quantification methods studied (Figure S6C and Table S6, Supporting Information). Additionally, Wemics standed out with the highest mean depth in DMRs, particularly stemming from unique Hypo‐Up and Hyper‐Down genes (Figure S7A,B, Supporting Information). Notably, ≈65% of Wemics‐detected DMRs associated with Hyper‐Down genes were located in promoters, which was significantly higher than those detected by other quantification methods (37% and *P* = 0.01 for Meanm; 42% and *P* = 0.004 for CHALM) (Figure 5C). These DEGs associated with Wemics‐detected DMRs were enriched for several cancer‐related pathways, such as MYC activity, regulation of epithelial cell proliferation, negative regulation of growth, and cell fate commitment (Figure 5D; Figure S8A, Supporting Information).

In addition, most of these Wemics‐detected DMRs in lung cancer were recurrently occurred in other primary cancers from TCGA, suggesting that Wemics‐detected DMRs are not only epigenetically conserved but also highly relevant to tumor development and progression (Figure S8B, Supporting Information). It is well known that tumor suppressor genes often become hyermethylated in cancer, resulting in downregulated gene expression (i.e., Hyper‐Down genes), while oncogenes become hypomethylated in cancer, resulting in upregulated gene expression^[^ ^7^, ^35^ ^]^ (i.e., Hypo‐Up genes). Therefore, we used the promoter methylation of these Hyper‐Down and Hypo‐Up genes as features to construct a classification model using the random forest algorithm. The DMR‐based methylation classifier could consistently distinguish tumors from adjacent normal tissues with AUCs exceeding 0.95 in most cancer types (Table S7, Supporting Information). Considering that TCGA data are unbalanced between positive and negative samples, we also calculated the specificity of the classifier, which represents the classification accuracy of negative samples (i.e., normal samples). As a result, the specificity was higher than 95% for all 15 types of primary cancer (Table S7, Supporting Information). These results reveal that methylation of the promoters of Wemics Hyper‐Down and Wemics Hypo‐Up genes is grossly aberrant during the development of various cancers, and that these aberrant alterations could serve as promising pan‐cancer diagnostic markers.

### 2.7 Methylation of TFBS Regulates Gene Expression

**Figure 6:**
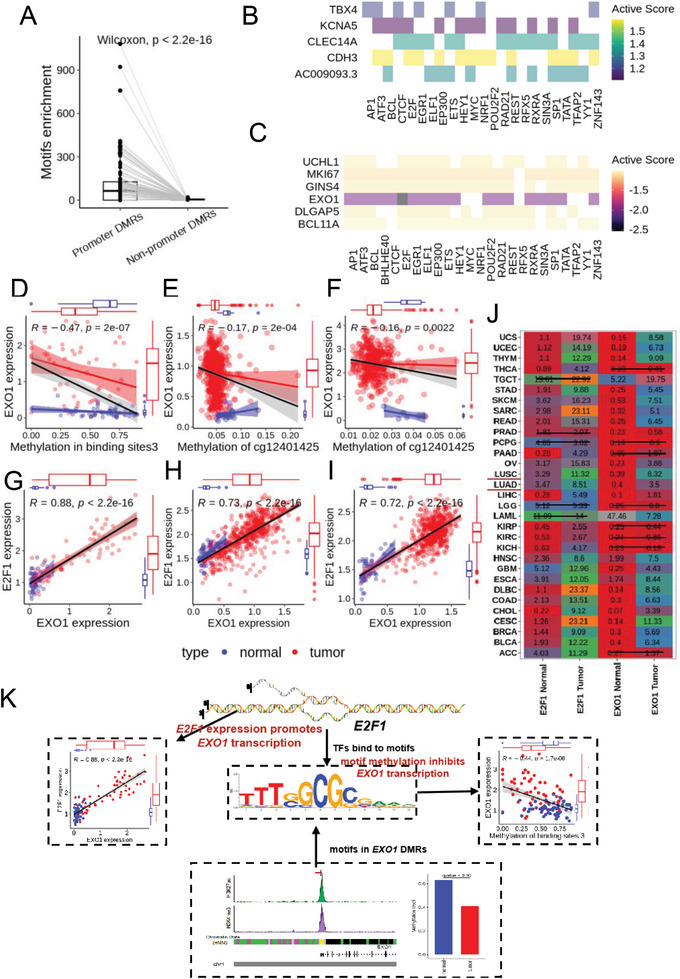
Methylation of transcription factor binding motifs affects their gene expression. A) Methylation‐sensitive motifs were preferentially presented in promoter DMRs in NSCLC. B,C) Activity plot integrating the correlation between methylation of TFBM and expressional fold changes of methylation‐induced genes. The strength of activation B) and inactivation C) of methylation‐induced genes in tumors compared to normal samples is indicated by color intensity. TFBM: transcription factor binding motifs. D) Correlation between methylation level of the E2F motif and expression level of EXO1 in our NSCLC samples. The Pearson correlation coefficient reached −0.47 (p‐value = 2e‐07). The black line represents the correlation curve fitted by both tumor (red) and normal (blue) samples and the 95% confidence interval of the Pearson correlation coefficient was plotted, and the R value was marked in the figure. E,F) Correlation between methylation level of cg12401425 and expression level of EXO1 in TCGA LUAD and LUSC datasets. cg12401425 is a CpG site located within the E2F motif. The Pearson correlation coefficient reached −0.17 (p‐value = 2.2e‐04) in LUAD and −0.16 (p‐value = 2.2e‐03) in LUSC. G–I) Correlation between E2F1 expression level and EXO1 expression level in our NSCLC samples (G), TCGA LUAD (H), and LUSC (I). E2F1 is a transcription factor regulating EXO1 expression. The expression of EXO1 and E2F1 showed a significant positive correlation in all three groups. The expression of EXO1 and E2F1 in Figure 6D–H were normalized to log2(FPKM+1). J) Expression levels of E2F1 and EXO1 in tumors and adjacent normal tissues of 31 cancer types in TCGA. Numbers with a strikethrough indicate no significant expression difference between tumors and adjacent normal tissues of that cancer type; otherwise, there is a signficant difference. The labeled numbers are Transcripts Per Million (TPM) of the gene. K) Schematic diagram of E2F1 regulating EXO1 expression. Decreased methylation in E2F motif within the EXO1 promoter can facilitate E2F1 binding, thereby increasing EXO1 expression. While increased expression in E2F1 can further promote EXO1 gene expression.

Transcription factor binding motifs (TFBMs) are a set of short recurring genomic sequences that specifically bind to transcription factors (TF). TFBMs are usually located in gene promoters with abundant CpG sites. Methylation of TFBM can alter its DNA structure, thereby affecting gene transcription by either inhibiting or promoting its binding to TF. Therefore, we evaluated whether these methylation‐sensitive TF motifs occur frequently in hyper‐ and hypo‐DMRs identified by Wemics in our NSCLC samples and whether methylation of these regulatory motifs influences expression of adjacent genes. As expected, methylation‐sensitive TF motifs were preferentially presented in promoter DMRs in NSCLC samples (**Figure** **6**A).

To further determine whether genes were strongly affected by the methylation status in the binding vicinity of the corresponding TFs in NSCLC, we calculated an activity score by integrating the correlation of targeted gene expression with the methylation level of TF motifs in DMRs. The activity score indicates the state of the target genes in tumor cells due to their (de)methylation of TF motifs; its absolute value indicates the degree to which the gene is activated (activity score > 0) or inactivated (activity score < 0). For an intuitive illustration, we presented target genes with the absolute value of activity score > 1.2 (Figure 6B,C). 24 significantly correlated motifs were identified in 11 target genes. Several well‐known methylation‐sensitive TFs such as *CTCF*, *SP1*, *TATA*, and *YY1* were also identified.^[^ ^36^ ^]^ The highly inactivated genes, whose corresponding TF binding motifs were significantly affected by DNA methylation, included several well‐known genes such as *TBX4* and *CLEC14A* and some novel genes such as *KCNA5* and *CDH3*. On the contrary, the strongly activated genes were also detected, such as *DLGAP5*,^[^ ^37^, ^38^ ^]^ *BCL11A*,^[^ ^39^ ^]^ *GINS4*,^[^ ^40^ ^]^ *EXO1*,^[^ ^41^ ^]^ *UCHL1*,^[^ ^42^ ^]^ and *MK167*,^[^ ^43^ ^]^ all of which had already been confirmed as potential oncogenes in lung cancer.

Among them, the *E2F* motifs in *EXO1* had the highest activity score. There were 19 E2F‐binding sites in 108‐bp DMR within the *EXO1* promoter. We merged these E2F‐binding based on their overlapping regions, resulting in three distinct, larger *E2F* motifs, *motif*_1_, *motif*_2_, and *motif*_3_ (Figure S9A, Supporting Information). The methylation level in each of these three merged motifs was significantly negatively correlated with *EXO1* expression in our lung cancer samples (Figure 6D; Figure S9B, Supporting Information). Of note, there was a CpG site (cg12401425) in the *motif*_3_ contained in the Illumina Infinium HumanMethylation450 BeadChip.^[^ ^44^ ^]^ Therefore, we analyzed Illumina methylation assays of lung adenocarcinoma (LUAD) and lung squamous cell caricinoma (LUSC) from TCGA, showing that the methylation of cg12401425 was significantly negatively associated with expression of EXO1 (Figure 6E,F). Both our and TCGA samples mutually validated that abnormally reduced methylation levels within the E2F motif are closely associated with the activation of EXO1 in NSCLC. Notably, this negative correlation was more pronounced in the tumor than normal samples. Specifically, for our NSCLC samples, the Pearson correlation between EXO1 expression and methylation in binding sites3 reached −0.27 (tumor), −0.22 (normal), and −0.47 (overall) (Figure 6D). This observation suggests that the impact of E2F motifs methylation on EXO1 expression tends to be more robust after tumor formation in lung cancer. Similar trends were also identified in TCGA LUAD and LUSC samples (Figure 6E,F).

E2F1, a major member of the E2F family, plays a crucial role in the control of cell cycle and action of tumor suppressor proteins. It had been shown that the downregulation of *E2F1* can induce repression of *MSH2, MSH6, EXO1*, and *RAD51*.^[^ ^45^ ^]^ We thus investigated the relationship between *EXO1* and *E2F1* expression levels and found their expression levels were also highly, positively correlated, not only in our lung cancer samples (Figure 6G) but also in LUAD and LUSC datasets from TCGA (Figure 6H,I). Surprisedly, in all 31 cancer types in TCGA except low‐grade glioma (LGG), EXO1 and E2F1 were consistently upregulated in tumor samples compared to normal samples, and the upregulation of expression of these two genes was statistically significant in 20 of these cancer types (Figure 6J). Taken together, *E2F1* is potentially involved in transcriptional regulation of *EXO1* and the reduced methylation of *E2F1* binding motifs may increase the ability of *E2F1* to bind to *EXO1*, thereby leading to the activation of *EXO1* in lung cancer (Figure 6K).

We then assessed to what extent the expression of the target gene was affected by the methylation level of the TFBS and the expression level of the corresponding TF. Here we took the transcription factor SETDB1 regulating its downstream target ZNF454 as an example. The methylation level in SETDB1 motif binding sites (SETB1_disc2, chr5:178941183‐178941192) within ZNF454 promoter was strongly inversely correlated with *ZNF454* expression (*r* = −0.52, *p* = 3e‐09, Figure S9C, Supporting Information). It is noteworthy that the negative correlation between methylation of SETDB1 motifs and ZNF454 expression was also stronger in tumor samples (*r* = −0.66, *p*‐value = 4.8e‐08) compared to normal samples (*r* = −0.37, p‐value = 5.97e‐03) (Figure S9C, Supporting Information). Additionally, a significantly positive correlation was observed between the expression of *SETDB1* and *ZNF454* (r = 0.27, *p* = 5.1e‐03, Figure S9D, Supporting Information). However, the positive correlation between the expression of ZNF454 and STEDB1 was more pronounced in normal (*r* = 0.75, p‐value = 4.6e‐02) than tumor (*r* = 0.27, p‐value = 5.1e‐03) samples. This discrepancy may due to the fact that ZNF454 expression (log2(FPKM+1)) is less variable in normal samples, primarily concentrated in the range of 0.3‐0.6 (Figure S9D, Supporting Information). Similar trends were discerned in the LUAD (Figure S9E,F, Supporting Information) and LUSC (Figure S9G,H, Supporting Information) from TCGA datasets.

A linear model was then constructed with the expression of *ZNF454* as the dependent variable and the methylation of SETDB1 binding motif and/or the expression level of SETDB1 as the independent variables using our lung cancer data (Table S8, Supporting Information). As a result, the methylation of SETDB1 binding motif and the expression of *SETDB1* accounted for 6% and 27% of the variation in *ZNF454* expression, respectively (adjusted R^2^ = 0.06 and R^2^ = 0.27). However, the two could jointly explain 40% of the variations in ZNF454 expression (adjusted R^2^ = 0.40), and if both and their interactions were considered together, they could further explain 45% of the variation in ZNF454 expression (adjusted R^2^ = 0.45). Consistent results for the regulation of ZNF454 expression by SETDB1 were observed in the TCGA LUAD and LUSC datasets (Tables S9 and S10, Supporting Information).

Among 315 Hyper‐Down and Hypo‐Up genes, at least 20% of the expression changes in 176 genes could be explained by the methylation levels of TFBM and the expression levels of the corresponding TFs and their interactions (Table S11, Supporting Information). These data suggest that DNA methylation and TF act in concert and in parallel to form a complex regulatory mechanism of gene expression.

## 3 Discussion

In this study, we proposed a new method for DNA methylation quantification at single‐base resolution, namely Wemics, which assigns weights to the methylation of consecutive CpG sites in a genomic region. By leveraging the information from neighboring CpG sites, which tend to have correlated methylation patterns, Wemics provides a reliable quantification of DNA methylation levels. It also reveals cell heterogeneity in DNA methylation patterns by distinguishing between methylated reads, unmethylated reads, and partially methylated reads in bulk sequenced cells.

Integration analysis of DNA methylome and transcriptome data from a large set of NSCLC samples showed that Wemics could capture a stronger negative correlation between the methylation levels of gene promoters/enhancers and their gene expression levels than other quantification methods such as CHALM and CAMDA. Consistently, Wemics‐quantified DNA methylation levels also exhibited stronger regulatory relationships with the intensities of histone marks such as H3K27ac, H3K4me3, and H3K27me3 in gene promoters. Therefore, DNA methylation levels quantified by Wemics better reflect their regulatory impact on gene transcription and expression.

DNA methylation quantification has a very important impact on downstream DMR identification. Compared with other DNA methylation quantification methods, Wemics detected a higher frequency of DMRs distributed on functionally important regulatory regions, such as CpG islands and promoters. Therefore, DMRs detected under Wemics quantification tended to have more potentials to regulate the expression of their associated genes. This was evidenced by the fact that under Wemics quantification, the detected DMRs contained the largest proportion of DEGs (55.9%), with Hypo‐Up (20.0%) and Hyper‐down (18.1%) genes accounting for the vast majority of DEGs. Importantly, over 65% of the DEGs with Wemics‐quantified DMRs have been linked to lung cancer in previous studies (Figure 5B; Table S6, Supporting Information). They were significantly involved in several cancer‐related pathways, such as MYC activity, which has been shown to be a versatile oncogene with prognostic and therapeutic implications.^[^ ^46^, ^47^ ^]^

It is also important to highlight the distinctive characteristics of each methylation quantification method in identifying DMRs. The emergence of unique DMRs detected by each method is largely attributed to its distinct focus. Specifically, CAMDA prioritizes unmethylated CpG sites on partially methylated reads, CHALM concentrates on reads featuring methylated CpG sites, Meanm emphasizes averaged methylated CpG sites, and Wemics targets consecutively methylated CpG sites with different weights. The diverse focal points inherently result in varying methylation levels. For the unique DMRs identified under each specific method, they exhibit the highest absolute level of methylation difference between tumor and normal groups under that particular quantification method. This observation, as depicted in Figure S7C (Supporting Information), underscores that these regions are more likely to be detected as DMRs due to their pronounced disparities in methylation levels. Interestingly, unique Wemics DMRs often exhibit consistently higher mean sequence depth when compared to other methods.

In addition, most Wemics‐quantified DMRs in lung cancer were epigenetically conserved and recurrently occurred in other primary cancers from TCGA. Classifiers using methylation of these promoter DMRs could accurately distinguish tumors from normal tissues in various cancer types. This illustrates that aberrant DNA methylation of Wemics Hyper‐Down and Hypo‐Up genes is a common epigenetic mechanism in the development of various cancers, and that these aberrant alterations could serve as promising pan‐cancer diagnostic markers (Table S7, Supporting Information).

One of the key mechanisms of carcinogenesis is the overexpression of oncogenes in tumor cells, which is often caused by Hypo‐DMRs in gene promoters.^[^ ^29^ ^]^ Wemics was more effective at finding Hypo‐DMRs than the other three methods. This led to the identification of 63 Hypo‐Up genes that were both upregulated and hypomethylated in NSCLC tumor tissues compared with adjacent normal tissues, many of which such as ODC1,^[^ ^31^ ^]^ BCL11A,^[^ ^32^ ^]^ CDCA7,^[^ ^33^ ^]^ and TRIM2^[^ ^34^ ^]^ were shown to play important roles in the development and progression of NSCLC, while others 19 novel Hypo‐Up genes such as *PCNX2, GOLGA7B, ZDHHC13*, and *MPP6* have not been reported in lung cancer. Studying these novel Hypo‐Up genes will reveal new pathways of epigenetic dysregulation in lung cancer, and shed light on new mechanisms of lung carcinogenesis. In contrast, the number of Hypo‐DMRs detected by other methylation quantification methods is relatively small, so DNA methylation quantification using these methods may miss key oncogenes regulated by Hypo‐DMRs in NSCLC.

TFs regulate gene expression by binding to specific DNA sequences (i.e., TFBMs) in the promoter of their target genes. The methylation status of TFBMs can change their DNA structure and affect how TFs bind to them, either enhancing or inhibiting gene expression. We found that DMRs‐associated DEGs in lung cancer were often influenced by both the methylation of TFBMs in their promoters and the expression levels of the corresponding TFs. A notable example was the E2F1 motif in the promoter of EXO1, which had a CpG site (cg12401425) that showed a strong negative correlation between its methylation level and the expression of EXO1 in our NSCLC and TCGA LUAD/LUSC datasets. Overall, out of 315 DMRs‐associated DEGs, more than 20% of the expression changes in 176 DEGs could be explained by the methylation levels of TFBM and the expression levels of the corresponding TFs and their interactions.

In summary, Wemics is an innovative approach for quantifying DNA methylation at single‐base resolution in RRBS data. Wemics quantification of DNA methylation enhances the prediction of its impact on gene transcription and identifies DMRs with heightened biological relevance. Using Wemics on epigenomic‐transcriptomic data from our large NSCLC cohort, we unveiled several genes with oncogenic potential that were upregulated by hypomethylation but remained undetected by other quantification methods. These findings increase our understanding of the epigenetic mechanism governing gene expression. Furthermore, our study, constituting one of the largest epigenome‐transcriptome sequencing endeavors in lung cancer, establishes a valuable epigenetic resource. This resource holds potential for identifying DNA methylation‐based diagnostic biomarkers, facilitating the development of cancer drugs for epigenetic therapies, and advancing our understanding of lung cancer pathogenesis.

However, certain study caveats merit acknowledgment. While Wemics excels in quantifying methylation at single‐base resolution, it has limitations in quantifying methylation on a single read, thereby constraining its applicability in certain scenarios. Notably, Wemics quantification bias may arise in regions with low CG content, especially when only a minimal number of CpGs are present on the sequencing reads (as seen in extreme cases like only one CpG). Under such circumstances, Wemics quantification fails to consider the weights of consecutively methylated CpG sites, limiting the full utilization of its quantification superiority. Further investigations are needed to assess Wemics' performance under varying sequencing parameters such as coverage depth and read length. In our analysis of sequencing depth's impact on methylation levels, we randomly sampled 80% and 90% of bam file data, focusing on CpG sites with pre‐sampling coverage ≥ 10, post‐sampling coverage ≥ 5, and a coverage difference ≥ 5. Results, except for CAMDA, showed greater centralization around 0 for other methods, highlighting their stronger stability and consistency (Figure S10A,B, Supporting Information). The study of depth changes for subsequent analyses is influenced by various factors, as previously examined for their effects on differentially methylated sites and regions.^[^ ^28^, ^48^ ^]^ The impact of these factors on Wemics' quantification of DNA methylation awaits further exploration.

## 4 Experimental Section

### RRBS and RNA‐Seq

Seventy‐seven lung tumors and matched adjacent normal tissues were collected from NSCLC patients (Table S1, Supporting Information). Fresh tissues were snap‐frozen in liquid nitrogen, and stored at −80 °C. Hematoxylin and eosin (H&E) slides were examined by a pulmonary pathologist to confirm the diagnosis. All tissue samples used in the current study were obtained with permission at the time of diagnosis, before any treatment was administered. The study protocol was approved by the Institutional Review Board of Sir Run Run Shaw Hospital at Zhejiang University School of Medicine (Hangzhou, China).

Genomic DNA of these tissues was extracted and then processed according to our previously described RRBS library preparation protocol with modifications to allow multiplexing.^[^ ^13^, ^48^ ^]^ Paired‐end sequencing with 150bp was performed on the Illumina Novaseq 6000 platform according to manufacturer's protocol (Table S2, Supporting Information). Unconverted cytosines at fill‐in 3’MspI sites of sequencing reads were used to estimate bisulfite conversion rate. For each CpG site, DNA methylation rate was calculated as the percentage of unconverted cytosines in each RRBS library. To demonstrate global methylation profiles in NSCLC samples, the top 1% of most varied CpG sites across all samples were selected to perform unsupervised hierarchical clustering and principal component analysis (PCA).

Total RNA from 55 of 77 pairs of NSCLC RRBS samples was prepared for mRNA sequencing libraries (Table S3, Supporting Information) as described previously.^[^ ^49^ ^]^ Detailed data processing and analysis of RRBS and RNA‐seq methods were described previously.^[^ ^28^, ^48^, ^50^ ^]^

### Wemics Quantifying DNA Methylation at Single‐Base Resolution

In this study, we proposed a single‐base resolution methylation quantification method by weighting methylation of consecutive CpG sites (Wemics) in genomic regions (Figure 1A). Wemics takes into account not only the methylation status of a given CpG site, but also the co‐methylation of its neighboring consecutive CpG site in the same sequenced read. Briefly, to quantify DNA methylation of a specific CpG site *S* in a RRBS library, we first retrieved all reads containing the CpG site *S* from the BAM file of a RRBS library. Next, these reads were grouped into two categories:

1) Methylated reads that contain at least one methylated CpG site, and the number of methylated reads is *n_m_*. Suppose the *i*‐th methylated read contains *c_i_* CpG sites (*i*  =  1, …, *n_m_*), among which there are *m_i_* consecutive methylated CpG sites. Then, the CpG site S has two different potential methylation states:
  i) The CpG site S is located within *m_i_* consecutive methylated CpG sites, and 1 ≤ *m_i_* ≤ *c_i_*.
  ii) The CpG site S is unmethylated, and set *m_i_* =  1.
2) Unmethylated reads that do not contain any methylated CpG sites, and the number of unmethylated reads is *n_u_*.

Finally, the methylation rate of the CpG site *S* in the RRBS library is quantified as,

(1) $$\mathrm{Wemic}s_{S}=\frac{\sum_{i=1}^{n_{m}}c_{i}m_{i}}{\sum_{i=1}^{n_{m}}c_{i}m_{i}+n_{u}}$$

For CHALM, the methylation rate of the CpG site *S* in the RRBS library is quantified as,

(2) $$\;\mathrm{CHAL}M_{S}=\frac{n_{m}}{n_{m}+n_{u}}$$

For CAMDA, assuming that  the number of unmethylated CpG site *S* in partially methylated reads is *n*_*p*, _ the methylation rate of the CpG site *S* in the RRBS library is quantified as,

(3) $$\mathrm{CAMD}A_{S}=\frac{n_{p}}{n_{m}+n_{u}}$$

For Meanm, the methylation rate of the CpG site *S* in the RRBS library is quantified as,

(4) $$\mathrm{Mean}m_{S}=\frac{n_{m}-n_{p}}{n_{m}+n_{u}}$$

The source code for implementing Wemics to quantify DNA methylation levels in RRBS data is available in the GitHub repository at https://github.com/sixone11/Wemics/tree/main.

### Correlations Between DNA Methylation Levels and other Regulatory Signals

Pearson correlation coefficients (*r*) were used to measure the linear correlation between the methylation level (*x_i_*) and other signal intensity (*y_i_*) such as gene expression and peak from ChIP‐seq.

(5) $$r=\frac{N\sum x_{i}y_{i}-\sum x_{i}\sum y_{i}}{\sqrt{N\sum x_{i}^{2}-{\left(\sum x_{i}\right)}^{2\;}\;}\sqrt{N\sum y_{i}^{2}-{\left(\sum y_{i}\right)}^{2}}}$$

where *i*  =  1, …, *N*, and *N* is the sample size.

Gene promoters were defined as 2kb upstream and downstream of the transcription start site (TSS). Enhancer annotations were downloaded from ENCODE.^[^ ^51^ ^]^ The closest enhancer within less than 2 kb of the TSS was assigned to the corresponding gene. Correlation between methylation levels at promoters or enhancers and corresponding gene expression levels was calculated using Pearson correlation coefficients. Gene expression was quantified by Fragments Per Kilobase Million (FPKM) and then normalized into log2(FPKM+1).

ChIP‐seq data of histone modification (such as H3K4me3, H3K27ac, and H3K27me3) from the A549 cell line were downloaded from ENCODE (http://hgdownload.cse.ucsc.edu/goldenPath/hg19/encodeDCC/wgEncodeBroadHistone).^[^ ^52^ ^]^ These downloaded ChIP‐seq data were previously aligned to human reference hg19 in the format of BAM file (https://www.encodeproject.org/pipelines/). Thus, we converted the aligned BAM file from hg19 to GRCh38 using the LiftOver pipeline (https://genome.sph.umich.edu/wiki/LiftOver). Then htseq‐count tool was applied to count mapped reads in promoter CGIs^[^ ^53^ ^]^ (v0.6.1p1, https://htseq.readthedocs.io/en/release_0.11.1/count.html). The signal intensity was normalized as,

(6) $$\;\mathrm{Signal}\;\mathrm{strength}=\;\log2\left(\frac{\#\;\mathrm{Read}\;\mathrm{count}\;\mathrm{in}\;\mathrm{promoter}\;\mathrm{CGI}}{\mathrm{promoter}\;\mathrm{CGI}\;\mathrm{length}*1000}+1\right)$$

To explore the methylation levels around some important regulatory modification peaks, we used deepTools (v3.1.3, https://deeptools.readthedocs.io/en/develop/) to normalize all peaks into 1kb and examined the methylation levels of 2kb upstream and downstream of the peaks.

### A Modified Metilene with Wilcoxon Signed‐Rank Test

Metilene was the preferred tool designed to detect DMRs in RRBS data.^[^ ^27^, ^28^ ^]^ It applies a binary segmentation algorithm to *de novo* identify genomic regions, maximizing methylation differences between two unpaired groups by the Mann‐Whitney U test. Since methylation data were from matched tumors and adjacent normal tissues of the same patient, we replaced the Mann‐Whitney U test by the Wilcoxon signed‐rank test:

(7) $$W=\sum_{i=1}^{N_{r}}\left[sgn\left(x_{2,i}-x_{1,i}\right)*R_{i}\right]$$

where *x*_1,*i*_ and *x*_2,*i*_ represents the methylation level at a CpG site from the i‐th patient's tumor and adjacent normal tissue, respectively; *R_i_* was the rank of the absolute methylation difference of site_i_ among all sites within this detected region.

### DMRs Identified under Different Methylation Quantification Methods

Prior to the DMR detection, each of the four quantification methods was used separately to quantify DNA methylation levels at CpG sites in our NSCLC RRBS data. We then applied the modified metilene to identify DMRs in 77 pairs of tumors and adjacent normal tissues from NSCLC patients. The minimum number of CpGs in a DMR was set to be 3, and the minimum mean methylation difference for calling DMRs was set to be 0.1. The detected DMRs were further classified as Hyper‐ and Hypo‐DMRs. Hyper‐DMRs are DMRs with higher average DNA methylation levels in tumor tissues than in their adjacent normal tissues, while Hypo‐DMRs are the opposite. In the study of commonly detected DMRs, two DMRs with a distance less than 200 bp are considered to be the same DMR.

### Functional Annotation of DMRs

To investigate the functional relevance of detected DMRs, the following annotated genomic features were comprehensively analyzed from three aspects: 1) the correlation between DMRs and genomic features; 2) the proportion of DMRs overlapping with genomic features; and 3) the distance of DMRs to genomic features.

CGI positions were downloaded through the UCSC table browser (https://genome.ucsc.edu/). CGI shore, CGI shelf, and inteCGI are adjacent genomic regions at different distances from CpG islands. Specifically, the CGI shore is defined as the region less than 2kb away from the CGI border; the CGI shelf is defined as the region between 2 and 4 kb from the CGI; and the inteCGI is defined as a region that is up to 4 kb away from a CGI (Figure S11, Supporting Information). Information on gene structures such as TSS, 3’/5’ UTR, exon and intron was obtained from the Genecode annotation (release40) (https://www.gencodegenes.org/human/).

The 15‐state chromatin of the A549 cell line was downloaded from RoadMap (https://egg2.wustl.edu/roadmap/web_portal/chr_state_learning.html), which was identified by multivariate hidden Markov model‐based ChromHMM (v1.10). Information on each chromatin state can be found in Table S4 (Supporting Information).^[^ ^54^ ^]^ Assay for Transposase‐Accessible Chromatin using sequencing (ATAC‐seq) of the A549 cell line was downloaded from GEO under accession GSE169955 (https://www.ncbi.nlm.nih.gov/geo/query/acc.cgi?acc = GSE169955). The broad histone modifications of the A549 ChIP‐seq were downloaded from ENCODE DCC (http://hgdownload.cse.ucsc.edu/goldenPath/hg19/encodeDCC/wgEncodeBroadHistone).

### Recurrent Analysis of DMRs Detected in NSCLC and other Cancer Types

To determine if these DMRs detected in NSCLC recur in other tumor types, we downloaded and analyzed Infinium Human Methylation450 array data containing 485,764 probes (each probe representing a CpG site) from TCGA for 21 primary cancer types.^[^ ^55^ ^]^ In these array data, beta values ranging from 0 to 1 were used to measure DNA methylation levels at each probe. DMRs detected in NSCLC were assigned their closest genes. These HM450 probes presented in either these DMRs or promoters of corresponding genes (+/− 2kb from TSS) were analyzed for their methylation changes between tumors and adjacent normal tissues of other cancer types. Furthermore, Hyper‐ and Hypo‐DMRs were analyzed separately for their recurrence in other cancer types.

### Identification and Analysis of DMRs‐DEGs Pairs

55 of 77 pairs of NSCLC RRBS samples also had RNA‐seq data, which were subsequently used to detect differentially expressed genes (DEGs) between lung tumors and adjacent normal tissues (false discovery rate, FDR < 0.05). There DEGs were further classified as Up‐ and Down‐regulated in tumors compared with adjacent normal tissues. To determine whether genes contained detected DMRs (including Hyper‐ and Hypo‐DMRs), we assigned the DMRs to the nearest genes that are less than 2 kb away from them. These DMRs‐associated genes were divided into five categories according to their methylation and expression level changes: Hyper‐Down, Hyper‐Up, Hypo‐Down, Hypo‐Up, and NS (not significantly differentially expressed between tumors and adjacent normal tissues). Among them, the Hyper‐Down and Hypo‐Up lists represent the well‐known inverse relationship between gene expression and its associated DNA methylation level, and are therefore prime candidates for methylation‐induced driver genes.

Gene Ontology (GO) analyses of these DMRs‐associated DEGs were conducted using Metascape (http://metascape.org).^[^ ^56^ ^]^ Furthermore, using promoter methylation of Hyper‐Down and Hypo‐Up genes as features, the R package randomForest (version 4.6.14) was used to build classifiers to distinguish tumors from normal samples for various cancer types in TCGA. The performance of classifiers was evaluated with tenfold cross validation in terms of accuracy, sensitivity, specificity, and precision.

### Analysis of impact of expression of TF and methylation of TFBF on the expression of targeted genes

Genomic coordinates of transcription factors (TF) and TF binding motifs (TFBM) were downloaded from ENCODE (http://compbio.mit.edu/encode‐motifs/),^[^ ^57^ ^]^ which contained binding motifs of 84 TF groups annotated from 427 human Chip‐seq data sets. DMR was extended 200bp more to its flanking regions. The motif occupancy rate (also termed motif enrichment score) was defined as the ratio of the total length of all individual motifs in a DMR to the total length of that DMR.

To determine whether genes are strongly influenced by methylation status in the vicinity of the corresponding transcription factors binding motif (TFBM) in NSCLC, we calculated activity scores by integrating the correlation of targeted gene expression with TFBM methylation levels in DMR with the differential expression of targeted gene between tumor and adjacent normal tissue:

(8) $$\mathrm{Activity}\;\mathrm{score}\;=\;r\times \mathrm{lo}g_{2}\left(\mathrm{fold}\;\mathrm{change}\right)$$

where *r* is the Spearman correlation coefficient between the TFBM methylation level in DMR and the expression of targeted gene, and fold change is the ratio of the expression of targeted gene in tumors to that in adjacent normal tissues. When multiple TFBM are presented in a DMR, the average activity score was calculated for the corresponding targeted gene. We only focused on genes whose DMR methylation levels are inversely correlated with their gene expression. By definition, the sign of activity score indicates the activation status of motif methylation in tumors compared to normal samples (<0 inactivated, >0 activated), and the absolute value indicates the activation strength.^[^ ^58^ ^]^

To further investigate the regulatory impact of TF expression and TFBM methylation on its target gene, we constructed multiple linear regression models with the expression of the target gene as the dependent variable and with the methylation within TFBM and the expression of TF and their interaction as the independent variables. Adjusted R^2^ for measuring goodness‐of‐fit of the regression models was estimated. After combining information of TFBS methylation and TF expression, the motif‐gene pairs with the largest adjusted R‐squared were selected for downstream analysis (Table S11, Supporting Information).

### Data Availability

Raw RRBS data used in this study were deposited in NCBI Sequence Read Achieve (https://www.ncbi.nlm.nih.gov/sra/) under accession number SRP125064 and in NGDC Genome Sequence Archive (https://ngdc.cncb.ac.cn/gsa/) under accession number HRA005509. 15‐state chromatin of the A549 cell line was downloaded from RoadMap (https://egg2.wustl.edu/roadmap/web_portal/chr_state_learning.html). The universal enhancer annotation was from ENCODE (https://www.encodeproject.org/annotations/ENCSR461KLY/). ATAC‐seq of the A549 cell line was downloaded from GEO under accession number GSE169955 (https://www.ncbi.nlm.nih.gov/geo/query/acc.cgi?acc = GSE169955). The gene annotation was downloaded from genecode (Release 40, GRCh38.p13) (https://www.gencodegenes.org/human/). The genomic coordinates of CpG island were downloaded through the UCSC table browser (https://genome.ucsc.edu/). The RNA‐seq and Illumine Human Methylation 450K data in TCGA were downloaded through UCSC Xena (https://xenabrowser.net/datapages/). For methylation data, the beta‐value files were downloaded. For RNA‐seq data, the FPKM files were downloaded. A549 ChIP‐seq for broad histone marks were downloaded from ENCODE DCC (http://hgdownload.cse.ucsc.edu/goldenPath/hg19/encodeDCC/wgEncodeBroadHistone). ENCODE transcription factors (TFs) were downloaded from ENCODE‐motifs browser (http://compbio.mit.edu/encode‐motifs/).

### Code Availability

The source code for implementing Wemics to quantify DNA methylation levels in RRBS data is available in the GitHub repository at https://github.com/sixone11/Wemics/tree/main.

## Conflict of Interest

The authors declare no conflict of interest.

## Author Contributions

Yi Liu and J.Y. contributed equally to this work. P.L., X.P., and Y. Lu considered and designed the study; Yi.Liu developed the algorithm for quantifying DNA methylation and performed data analysis. J.Y. conducted the preparations for RRBS libraries. J.L. conducted the preparations for RNA‐seq libraries. E.C. and J.Z. performed the pathological analysis of tumor tissues. W.P. and E.C. collected tissue specimens and clinical data. Y. Liu and M.L. helped to develop the protocol for preparing RRBS libraries. L.Z. provided technical support for high performance computation. Yi Liu, Y. Lu and P.L. wrote the manuscript. P.L., X.P., Y. Lu, H.X., X.L., Y. Liu, M.L. and H.Z. revised and commented the manuscript. All authors discussed and commented upon the study.

## Supporting information

Supporting Information

Supplemental Tables S1–S11

## Data Availability

The data that support the findings of this study are openly available in Genome Sequence Archive at https://ngdc.cncb.ac.cn/gsa/, reference number 5509.
