# Supplementary material for: Wemics: A Single‐Base Resolution Methylation Quantification Method for Enhanced Prediction of Epigenetic Regulation
Source: Adv Sci (Weinh). 2024 Mar 28;11(21):2308884. doi: 10.1002/advs.202308884 (PMC11151077; doi:10.1002/advs.202308884)
Supplement: Supplementary file 1 — Supporting Information [file ADVS-11-2308884-s002.pdf]

ADVANCED SCIENCE
Open Access

Supporting Information

for *Adv. Sci.*, DOI 10.1002/advs.202308884

Wemics: A Single-Base Resolution Methylation Quantification Method for Enhanced Prediction of Epigenetic Regulation

Yi Liu, Jiani Yi, Pin Wu, Jun Zhang, Xufan Li, Jia Li, Liyuan Zhou, Yong Liu, Haiming Xu, Enguo Chen, Honghe Zhang, Mingyu Liang, Pengyuan Liu, Xiaoqing Pan* and Yan Lu**

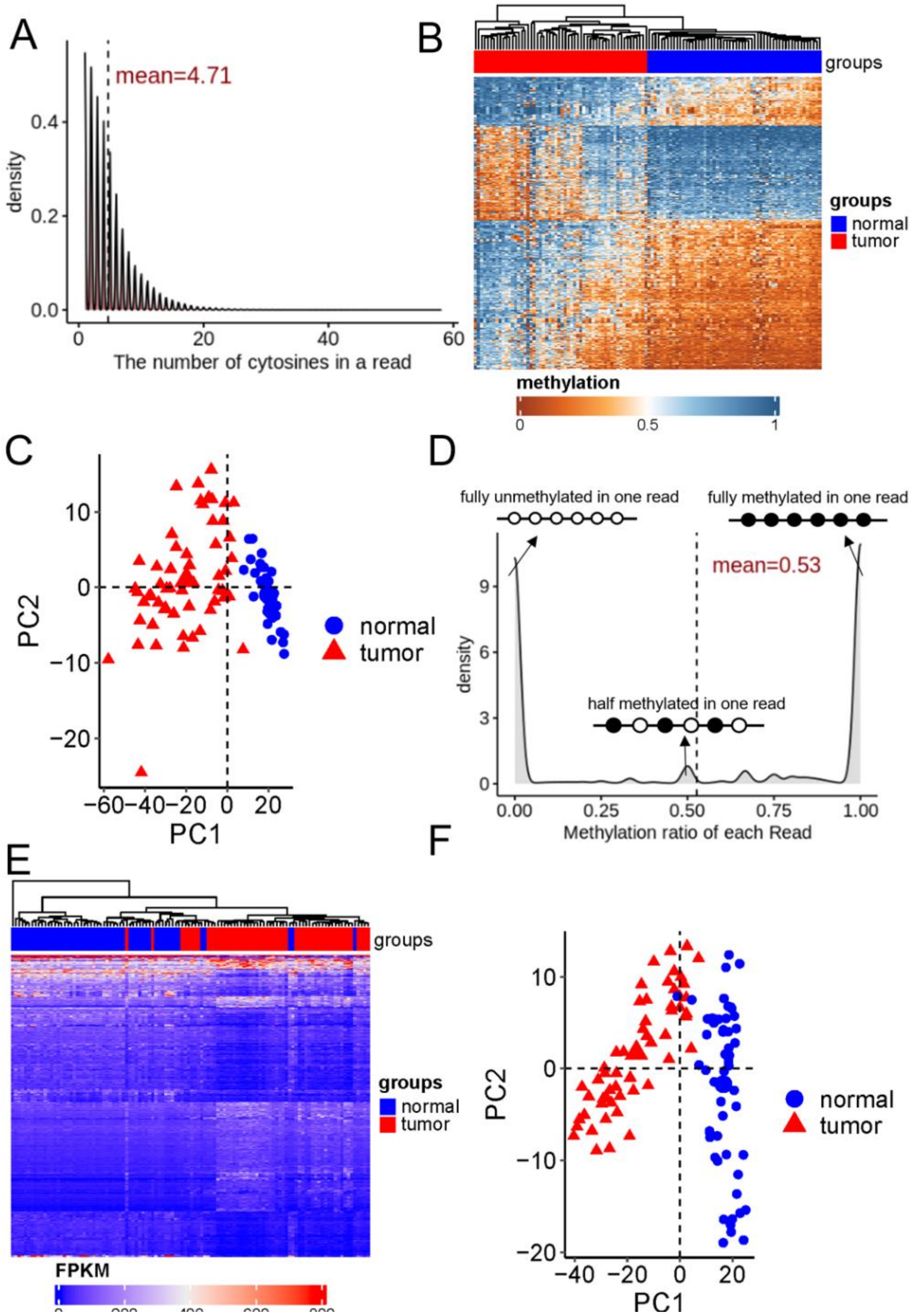

Figure S1 Summary of the results of RRBS and RNA-seq of NSCLC samples.

(**A**) Distribution of cytosine counts in sequenced reads. The average cytosine count of the sequenced reads is approximately 5.

(**B**) Unsupervised hierarchical clustering based on methylation levels of the top 1% CpG sites that varied most across tumors and adjacent normal tissues of NSCLC patients. Columns are samples and rows are CpG sites.

(**C**) Principal component analysis of 77 pairs of tumors and adjacent normal tissues of NSCLC patients based on methylation levels of all CpG sites.

(**D**) Distribution of methylation levels of sequenced reads. The highest densities were for fully unmethylated and fully methylated reads, followed by reads with half of the cytosine on the read being methylated.

(**E**) Unsupervised hierarchical clustering based on gene expression levels from RNA-seq data. Columns are samples and rows are genes.

(**F**) Principal component analysis of 55 pairs of tumors and adjacent normal tissues of NSCLC patients based on expression of all genes.

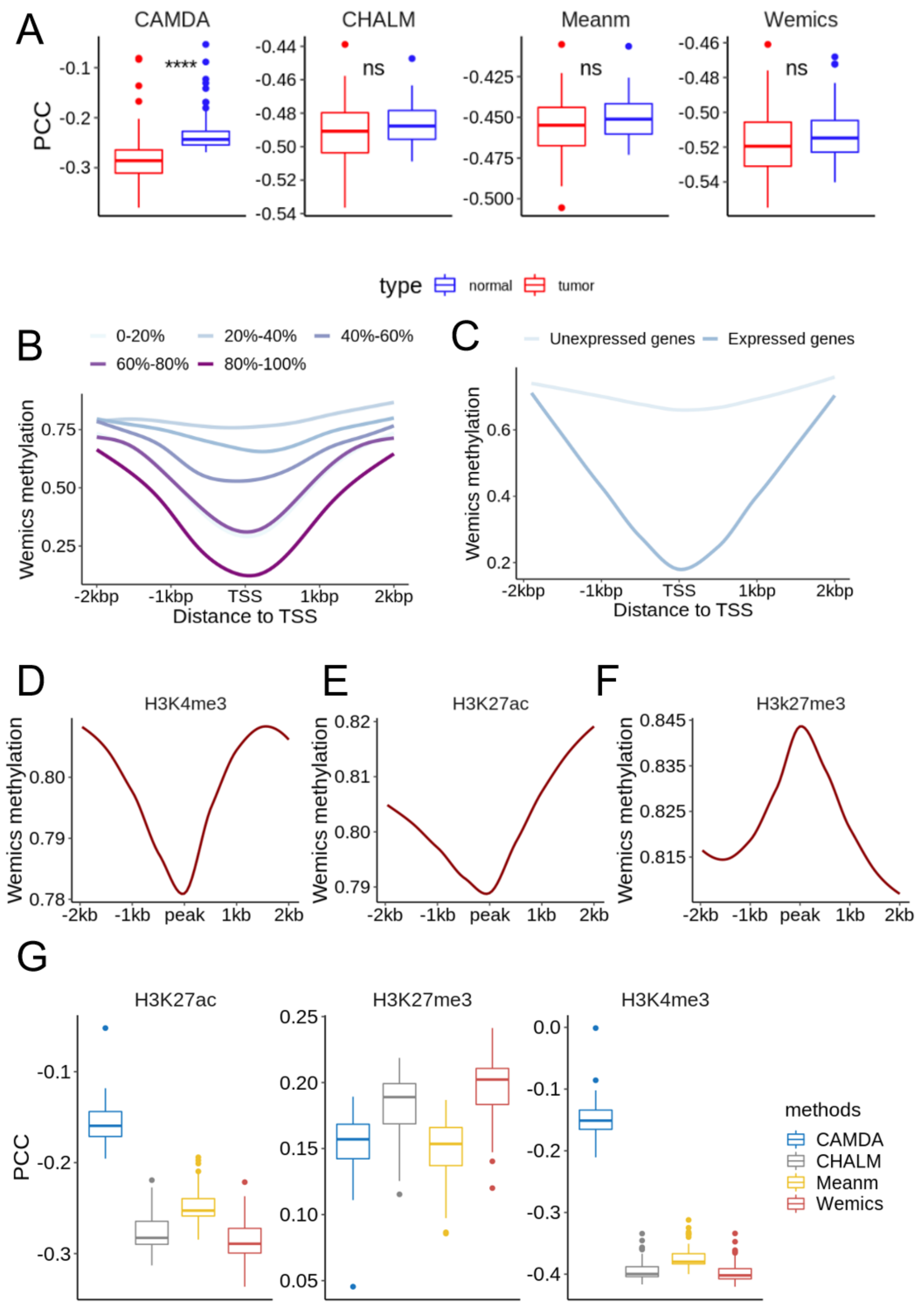

Figure S2. Relationship of DNA methylation with several regulatory signals. Related to Figure 2.

(**A**) Pearson correlation of promoter methylation and its gene expression in tumors and adjacent normal tissues of NSCLC samples. No difference in Pearson correlation between lung tumors and adjacent normal tissue except for CAMDA.

(**B**) DNA methylation levels across 2kb up- or downstream of TSS and their relationship with mRNA abundance. Genes were divided into five groups according to their expression levels, with 0-25% being the least expressed genes and 75-100% being the most expressed gene groups. TSS had the lowest level of methylation, with symmetrical increases on both sides. This phenomenon became more pronounced as gene expression increased.

(**C**) DNA methylation levels at 2kb upstream or downstream of the TSS for expressed and unexpressed genes. Hypomethylation of the TSS was still observed regardless of whether the gene was expressed or not.

(**D, E, F**) DNA methylation levels at 2kb upstream or downstream of peaks of histone modifications (H3K4me3, H3K27ac and H3K27me). For H3K4me3 and H3K27ac, the methylation level was lowest at the peak and increased symmetrically with the distance from the peak. For H3K27me3, the methylation level is highest at the peak and decreases symmetrically with the distance from the peak.
(**G**) Pearson correlation of H3K27ac, H3K27me3 and H3K4me3 signal intensity and gene expression when considering all genes on the genome. When we analyzed all genes on the genome, for H3K27me3, Wemics leads in terms of Pearson correlation, with a value of about 0.22, but other methods are less than 0.2. Wemics leads in terms of Pearson correlation for H3K27ac, with a value of about -0.29, and -0.4 for H3K4me3.

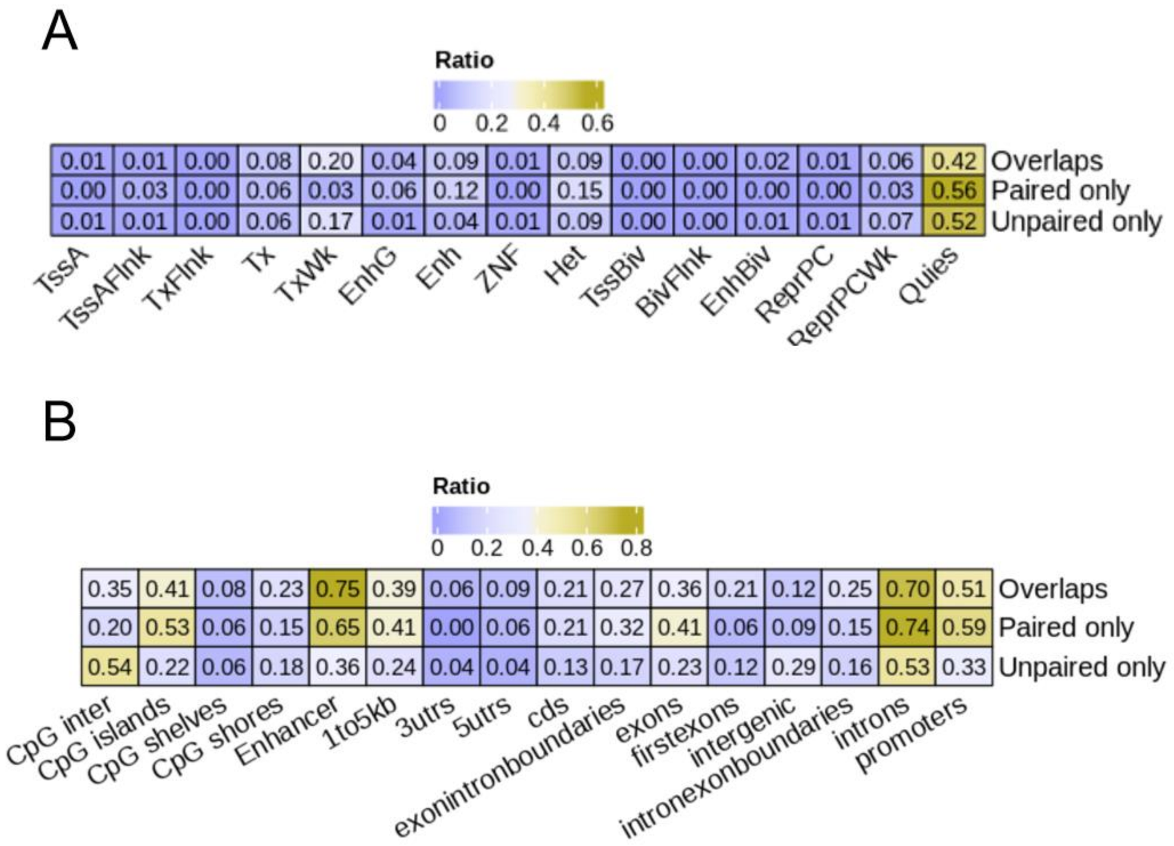

Figure S3 The intersection of DMRs detected by paired and unpaired metilene. Related to Figure 3.

(**A**) Proportional distribution of detected DMRs in 15 chromatin states of A549 cell line.

(**B**) Proportional distribution of detected DMRs in major functional regions of the genome.

Paired metilene with Wilcoxon signed-rank test and unpaired metilene with two-dimensional Kolmogorov–Smirnov test were respectively applied to detect DMR between tumors and matched adjacent normal tissues of NSCLC samples.

For a particular chromatin state or genomic feature, the ratio is defined as the number of DMRs with that chromatin state or genomic feature divided by the total number of DMRs.

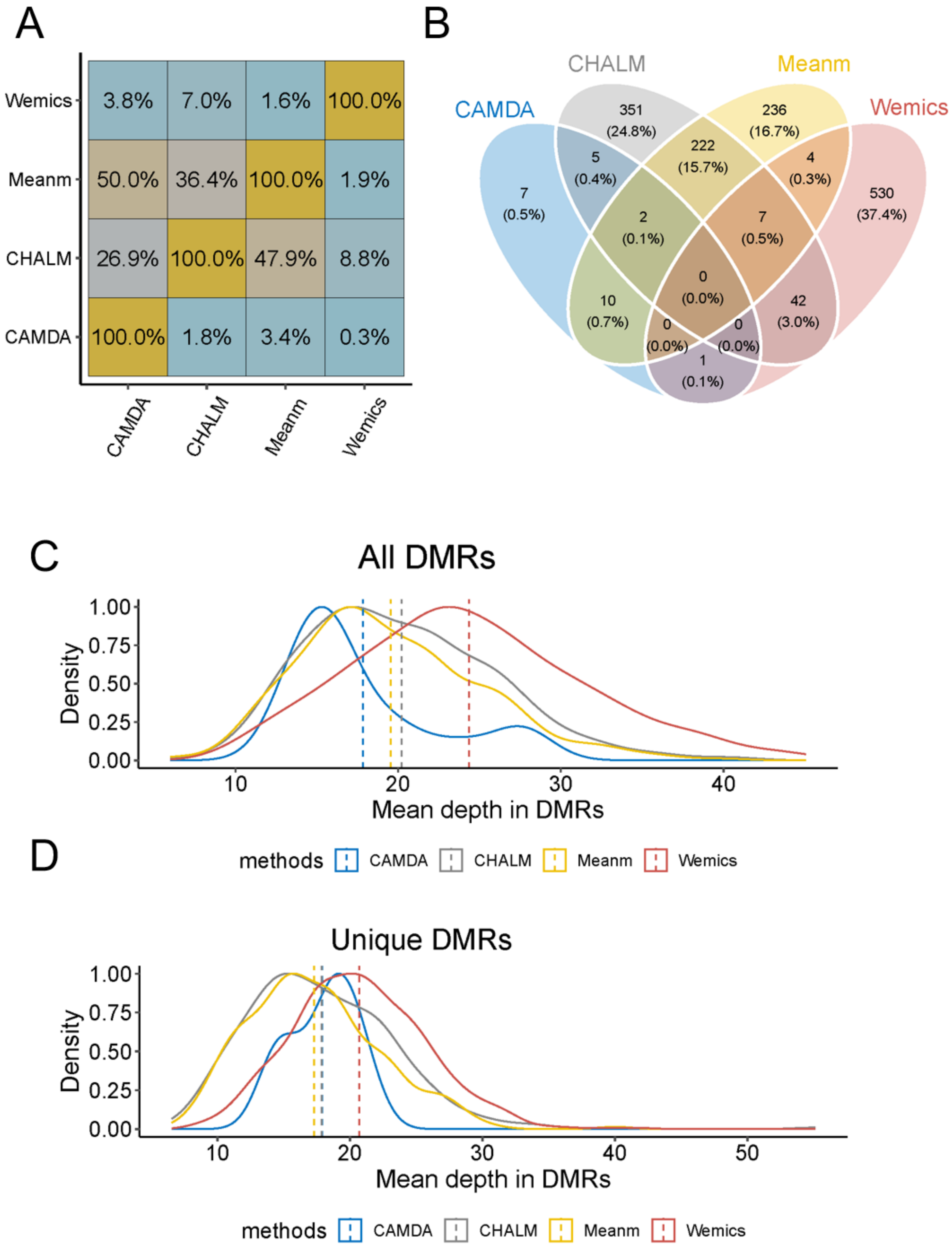

Figure S4 Overlap exploration of DMRs detected under different quantification methods. Related to Figure 4.
(**A**) Fraction of commonly detected DMRs under any two methylation quantification methods. Each cell represents the ratio of the number of commonly detected DMRs to the total number of DMRs detected under the respective quantification method in the vertical line.

(**B**) Venn diagrams of DMRs detected under four methylation quantification methods. We merged overlapping DMRs detected by different quantification methods, resulting in 1417 regions.
(**C**) Density plot of average sequencing depth within all DMRs detected under the four methylation quantification methods. The vertical line represents the mean value of the depth distribution.
(D) Density plot of average sequencing depth within unique DMRs detected by each methylation quantification method. The vertical line represents the mean value of the depth distribution.

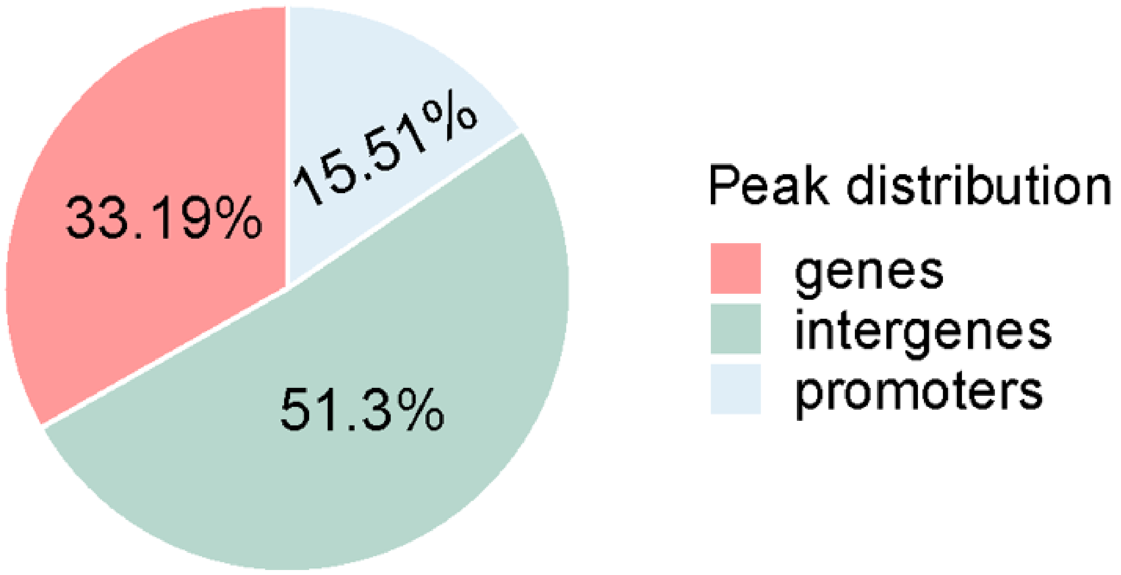

Figure S5 Distribution of ATAC-seq peaks of the A549 cell line. Related to Figure 4.

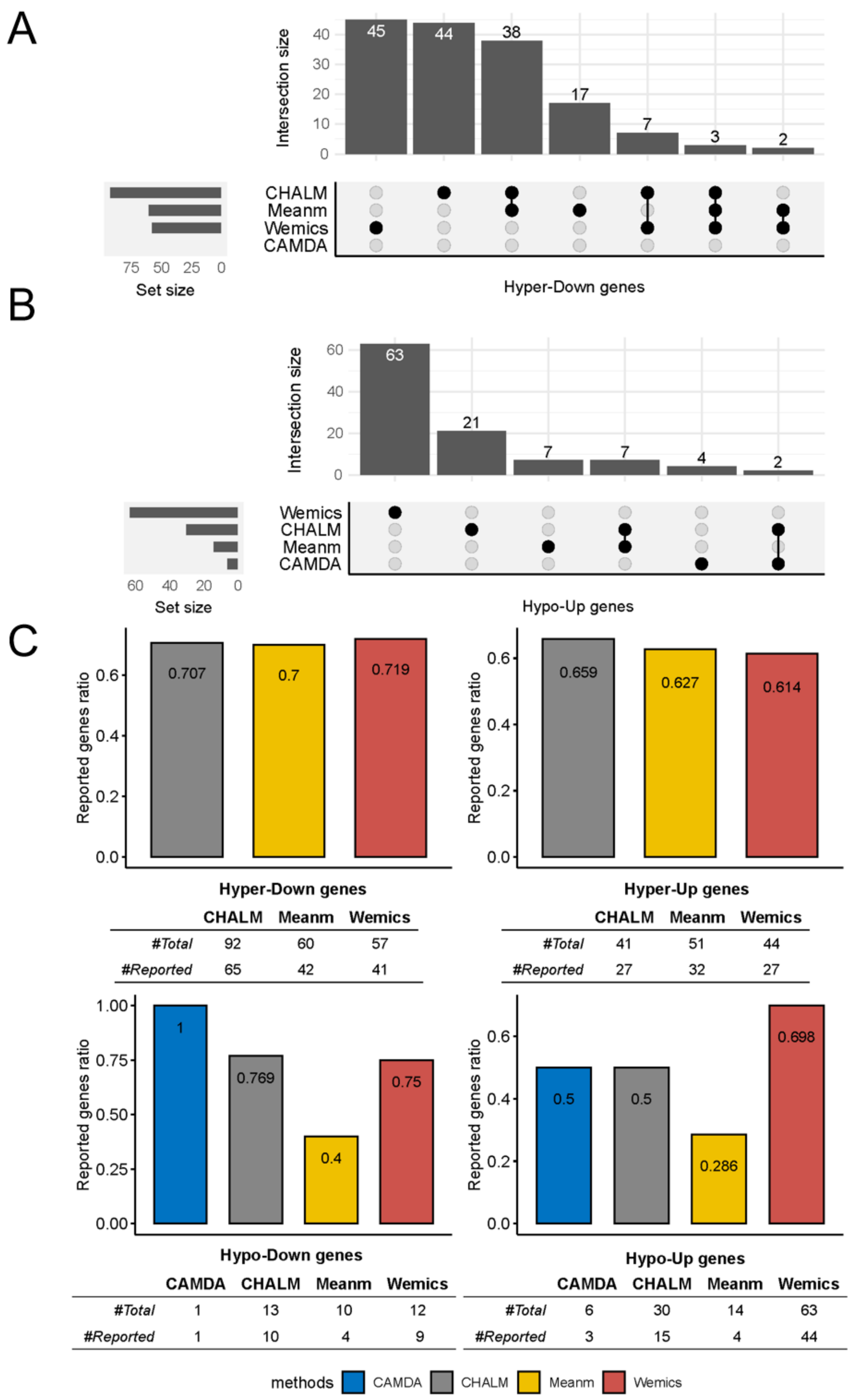

Figure S6 Relationships of DMRs-DEGs identified under four different methylation quantification methods. Related to Figure 5.

(**A**) The upset plots of Hyper-Down DMRs-DEGs determined by four different quantification methods.

(**B**) The upset plots of Hypo-Up DMRs-DEGs determined by four different quantification methods.

(**C**) The proportion of DMRs-DEGs that have been reported in previous studies to be strongly associated with lung cancer development and progression. We searched studies on genes and lung cancer through Malacards (https://www.malacards.org/). Please note no hyper-down genes were detected by CAMDA.

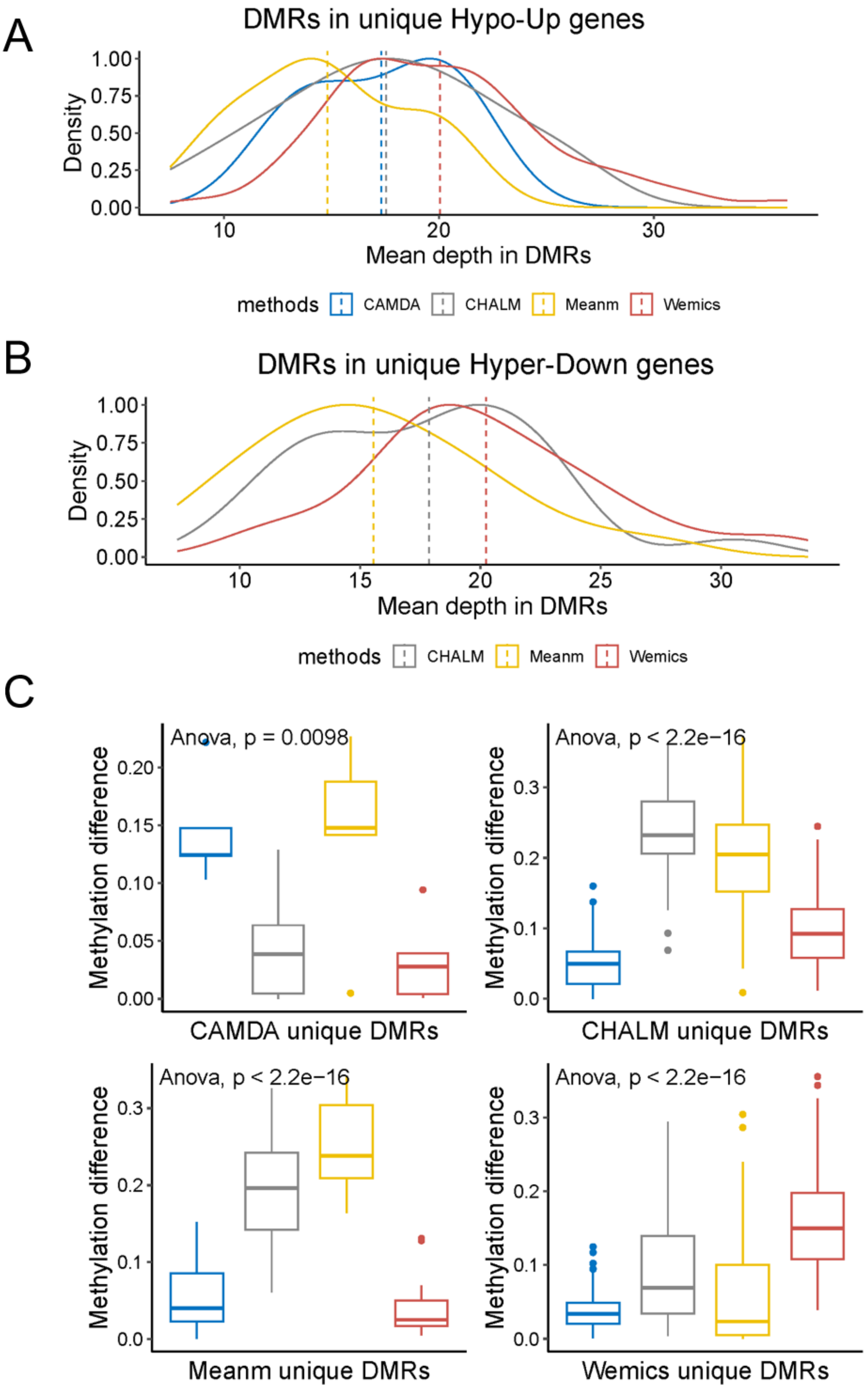

Figure S7 Characteristics of DMRs corresponding to unique DMRs-DEGs. Related to Figure 5.

(**A**) Density distribution of mean depth within DMRs corresponds to unique Hypo-Up DMRs-DEGs under different methylation quantification methods. Vertical lines represent the mean value of depth distribution.

(**B**) Density distribution of mean depth within DMRs corresponds to unique Hyper-Down DMRs-DEGs under different methylation quantification methods.

(**C**) Methylation difference within DMRs associated with Hyper-Down and Hypo-Up genes under a specific method.

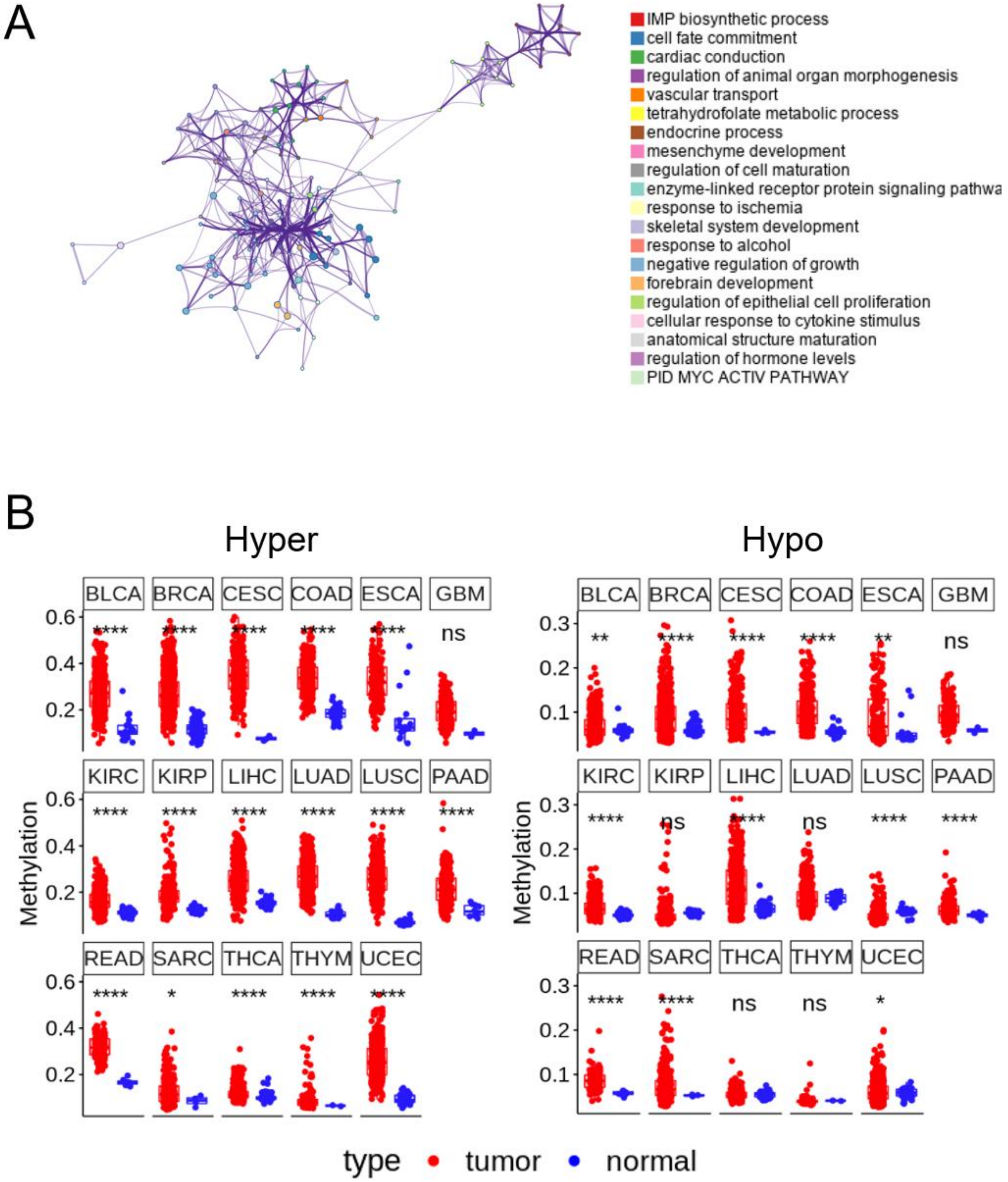

Figure S8 Additional properties of Wemics-quantified DMR detected in NSCLC. Related to Figure 5

(**A**) Predicted biological processes involved in hyper-Down and hypo-Up genes.

(**B**) Wemics-quantified DMRs detected in NSCLC were recurrent in other primary cancers from TCGA.

(**C**) ROC Plots of Sensitivity and Specificity of classifiers to distinguish tumors from adjacent normal tissues. A random forest tree algorithm was used to construct the classifier, which features aberrant methylation of Hyper-Down and Hypo-Up genes detected under Wemics quantification in our NSCLC.

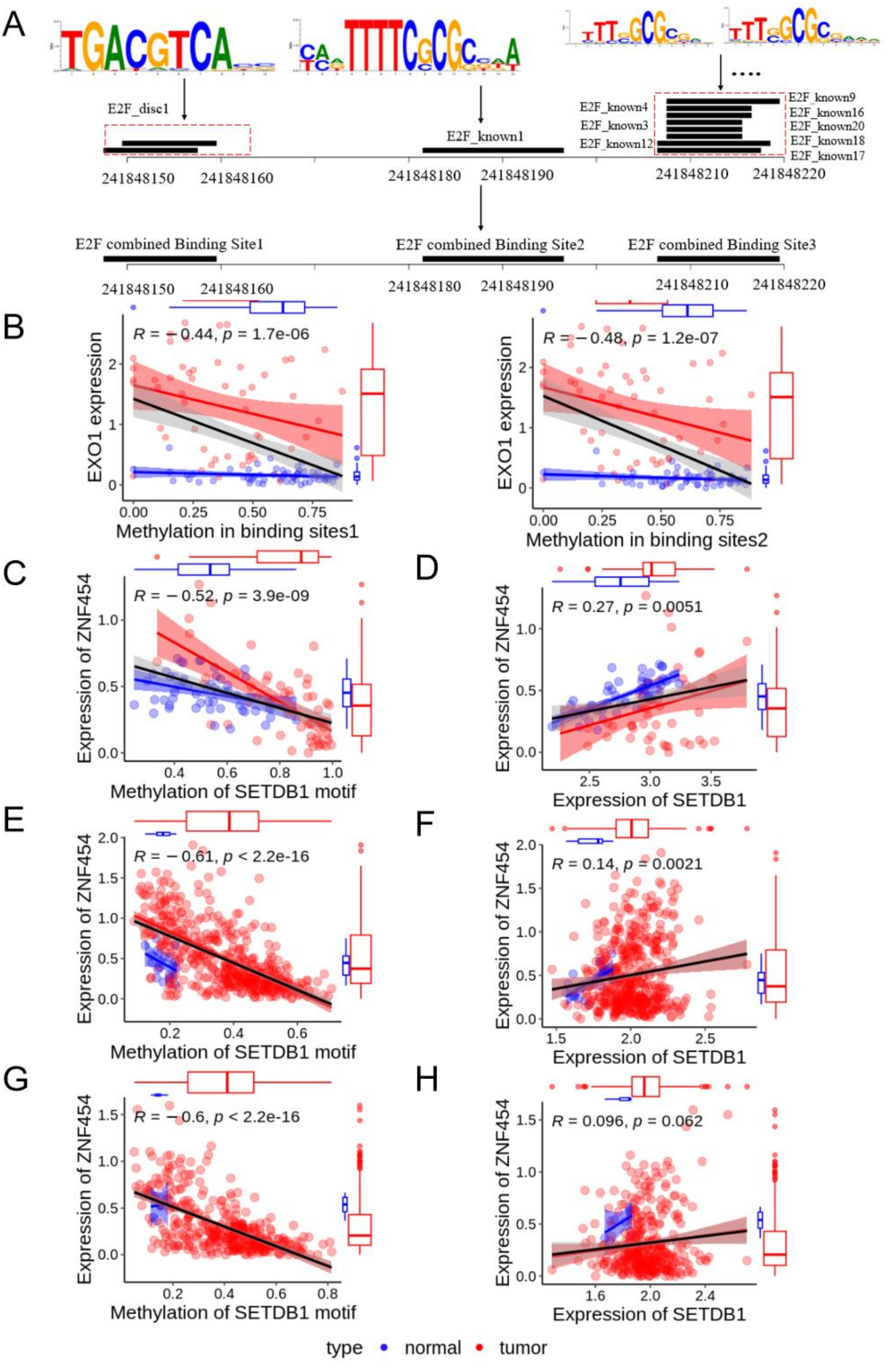
A
E2F_disc1
E2F_known1
E2F_known4
E2F_known3
E2F_known12
E2F_known9
E2F_known16
E2F_known20
E2F_known18
E2F_known17
241848150 241848160 241848180 241848190 241848210 241848220
E2F combined Binding Site1
E2F combined Binding Site2
E2F combined Binding Site3
B
R = −0.44, p = 1.7e-06
EXO1 expression
Methylation in binding sites1
R = −0.48, p = 1.2e-07
EXO1 expression
Methylation in binding sites2
C
R = −0.52, p = 3.9e-09
Expression of ZNF454
Methylation of SETDB1 motif
D
R = 0.27, p = 0.0051
Expression of ZNF454
Expression of SETDB1
E
R = −0.61, p < 2.2e-16
Expression of ZNF454
Methylation of SETDB1 motif
F
R = 0.14, p = 0.0021
Expression of ZNF454
Expression of SETDB1
G
R = −0.6, p < 2.2e-16
Expression of ZNF454
Methylation of SETDB1 motif
H
R = 0.096, p = 0.062
Expression of ZNF454
Expression of SETDB1
type normal tumor

Figure S9 TFBS Methylation regulates the expression of target genes. Related to Figure 6.

(**A**) TFBM of E2F in EXO1 DMRs. There are 11 different E2F binding motifs within the EXO1 DMRs. We merged these motifs with overlaps into one large region, yielding three merged motifs.

(**B**) Methylation in the other two merged E2F motifs was also negatively correlated with EXO1 expression in 55 pairs of NSCLC samples (Pearson' r < -0.44 (p-values =1.7e-06) and Pearson' r < -0.48(p-values <1.2e-07) for the two merged E2F motifs). The black line represents the correlation curve fitted by both tumor (red) and normal (blue) samples and the 95% confidence interval of the Pearson correlation coefficient was plotted, and the r value was marked in the figure.

(**C**) Correlation of ZNF454 expression with methylation of SETDB1-binding motifs within the ZNF454 promoter in our NSCLC samples. Pearson r is -0.52 and p-value is 3.9e-09.

(**D**) Correlation between expression levels of ZNF454 and SETDB1 in our NSCLC samples. Pearson r is 0.27 and p-value is 5.1e-03.

(**E**) Correlation of ZNF454 expression with methylation of SETDB1-binding motifs within the ZNF454 promoter in TCGA LUAD. Pearson r is -0.61 and p-value < 2.2e-16.

(**F**) Correlation between expression levels of ZNF454 and SETDB1 in TCGA LUAD. Pearson r is 0.14 and p-value is 2.1e-03.

(**G**) Correlation of ZNF454 expression with methylation of SETDB1-binding motifs within the ZNF454 promoter in TCGA LUSC. Pearson r is -0.60 and p-value < 2.2e-16.

(**H**) Correlation between expression levels of ZNF454 and SETDB1 in TCGA LUSC. Pearson r is 0.096 and p-value is 0.062. Expression of all genes in the graph was normalized to log2(FPKM+1).

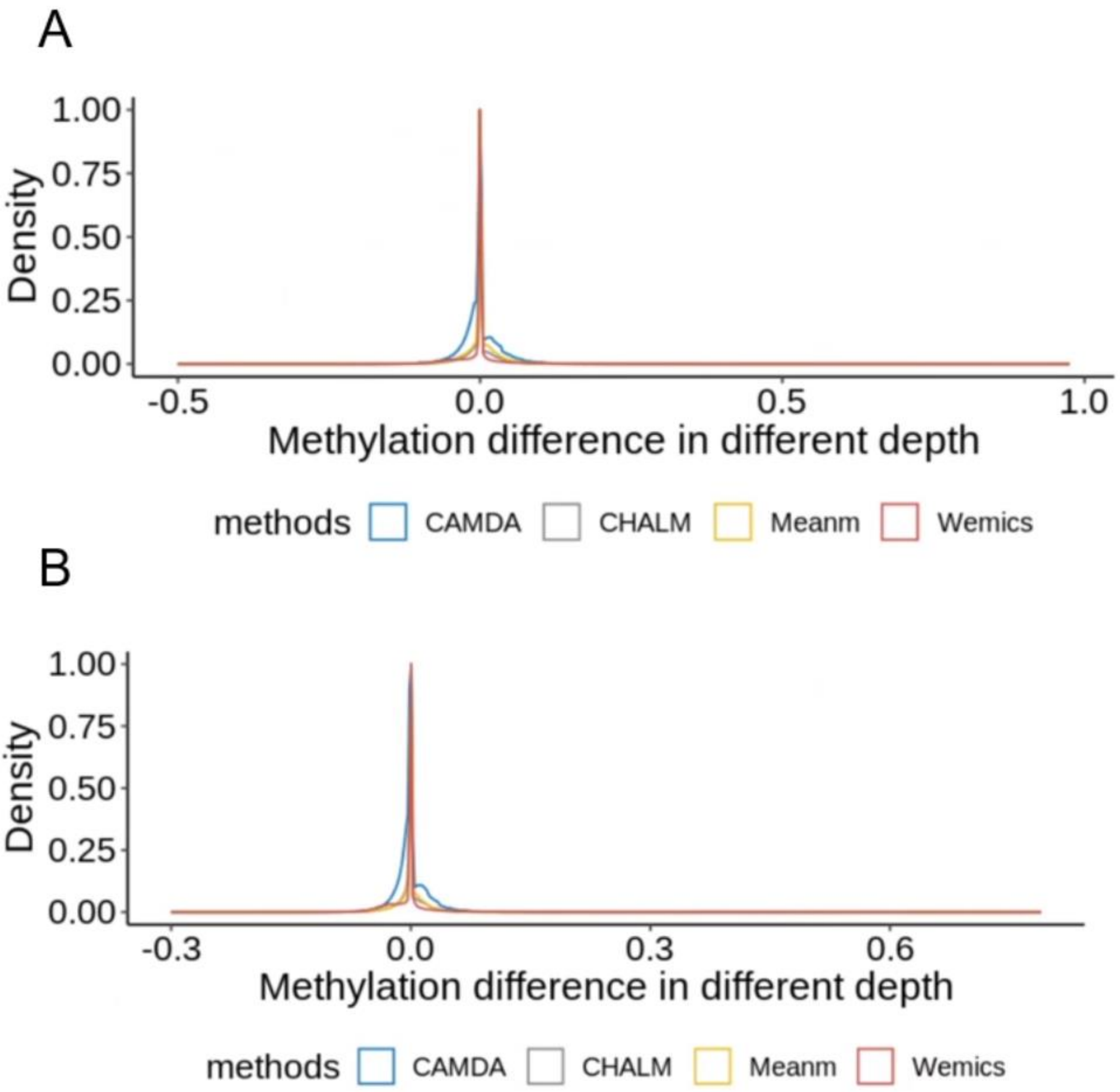

Figure S10. The methylation difference distribution of four quantification methods with randomly sampled data.

We randomly sampled 80% (A) and 90% (**B**) of the sequencing data (from bam file). The CpG sites whose pre-sampling coverage >= 10, post-sampling coverage is still greater than or equal to 5, and the difference in coverage between the pre- and post-sampling >= 5 are taken into consideration. The methylation difference is defined as pre-methylation minus post-methylation level in a CpG site.

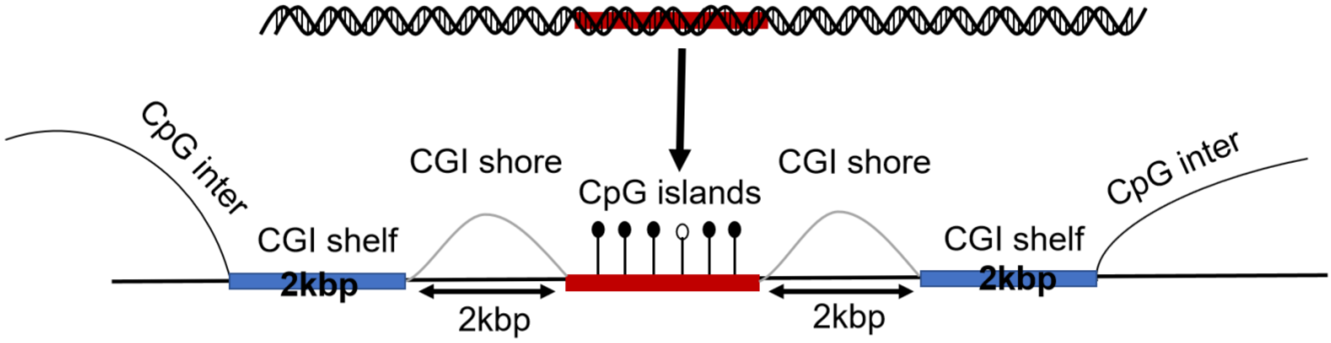

Figure S11. Defination of CGI shore, shelf and inter.

CGI positions were downloaded through the UCSC table browser (https://genome.ucsc.edu/). CGI shore, CGI shelf, and CGI inter are adjacent genomic regions at different distances from CpG islands. Specifically, the CGI shore is defined as the region less than 2kb away from the CGI border; the CGI shelf is defined as the region between 2 and 4 kb from the CGI; and the CGI inter is defined as a region that is up to 4 kb away from a CGI.
